# Electrifying Nitrogen Fixation: Plasma-Driven NO_*x*_ Synthesis for Sustainable Fertilizer Production

**DOI:** 10.1021/jacsau.5c01136

**Published:** 2025-11-20

**Authors:** Weitao Wang, Yaolin Wang, Hanwei Li, Michael Craven, Xin Tu

**Affiliations:** Department of Electrical Engineering and Electronics, School of Engineering, 4591 University of Liverpool, Liverpool L69 3GJ, U.K.

**Keywords:** plasma electrification, plasma catalysis, power-to-X, nitrogen fixation, ammonia, nitric acid

## Abstract

Decarbonizing nitrogen fixation is essential for sustainable fertilizer production, as the conventional Haber–Bosch process remains highly energy-intensive and a significant contributor to global greenhouse gas emissions. Plasma electrification offers a fossil-free, electricity-driven, and decentralized modular alternative that can operate flexibly with intermittent renewable energy sources. In this Perspective, we critically examine the current progress in plasma-based NO_*x*_ synthesis, with particular emphasis on reactor engineering, plasma–catalyst synergy, and plasma–liquid systems. We discuss how key operating parameters and plasma-induced reaction pathways govern efficiency and selectivity, and highlight recent advances that enhance NO_*x*_ yield while reducing energy consumption. Furthermore, we outline forward-looking strategies to improve plasma–gas interactions, suppress backward reactions, develop robust catalysts stable under nonequilibrium conditions, advance in situ diagnostics, and perform comprehensive techno-economic and life-cycle analyses to enable scalable and practical implementations. By highlighting these opportunities, this Perspective positions plasma-enabled nitrogen fixation as a transformative complement to the Haber–Bosch process, offering a sustainable route to fertilizer production that reduces fossil fuel dependence and mitigates environmental impact.

## Graphical abstract

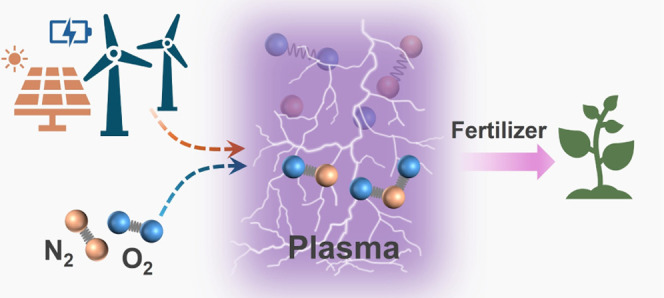

## 1 Introduction

### 1.1 Introduction to Nitrogen Fixation

Nitrogen is a fundamental element for all living organisms. It makes up about 78.08% of Earth’s atmosphere in the form of chemically inert diatomic N_2_ molecules. Converting atmospheric N_2_ into reactive nitrogen species, such as ammonia (NH_3_) and nitrogen oxides (NO_*x*_), is essential for biological and industrial nitrogen utilization. This transformation process is known as nitrogen fixation (NF). Naturally, atmospheric N_2_ can be captured and gradually converted by certain microorganisms through biological nitrogen fixation. However, the natural supply of fixed nitrogen compounds is insufficient to meet the increasing demand for ammonia and nitric oxide, particularly for fertilizer production. This gap has driven the development of large-scale industrial nitrogen fixation methods.

The Haber–Bosch (H–B) process for ammonia production remains the most crucial industrial nitrogen fixation process, supplying food for nearly half of the world’s population.^1^ Today, over 90% of global ammonia is produced via the H–B process, which depends heavily on fossil fuels.^2–4^ Although the conventional H–B process is highly energy-intensive, natural gas-based implementations remain the most cost-effective among current industrial methods and serve as the benchmark for emerging alternatives. A simplified configuration of a traditional natural gas-based H–B plant is shown in Figure 1. Despite the success of the H–B process, this process is associated with considerable greenhouse gas emissions. A modern natural gas-fed H–B plant releases ∼1.5–1.6 tons of CO_2_ equivalent per ton of ammonia, contributing to ∼1.2% of global anthropogenic CO_2_ emissions.^5,6^ These emissions originate mainly from the use of natural gas as a feedstock for hydrogen production rather than as a fuel.^7^ Bicer et al. demonstrated that switching from natural gas-based hydrogen production to hydropower electrolysis (the electrolysis-based H–B process in Figure 1) can reduce CO_2_ emissions by 75%, from 1.5 to 0.38 tons of CO_2_ equivalent per ton of ammonia.

**Figure 1:**
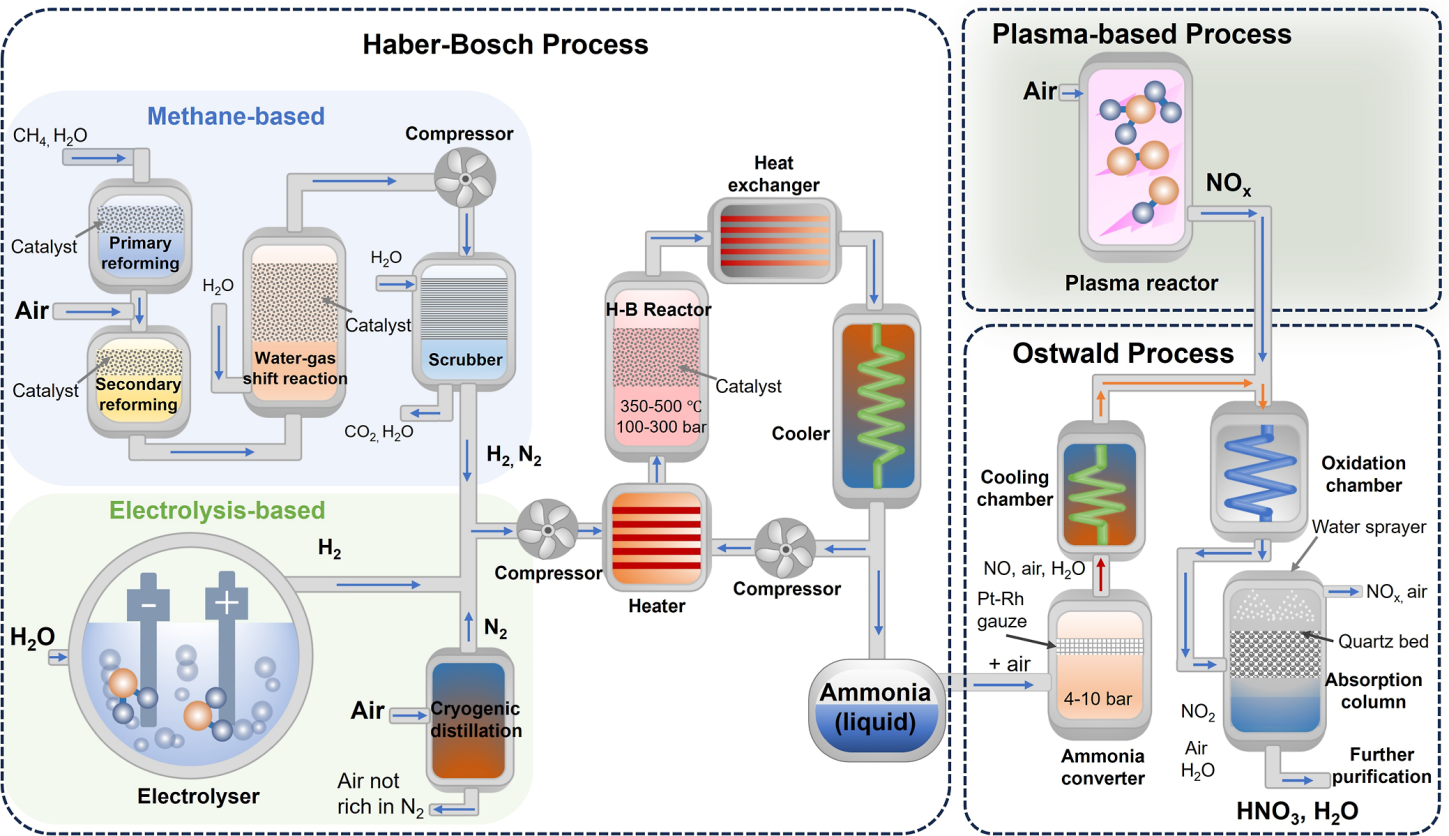
Schematic diagram of the methane-based and electrolysis-based H–B (e H–B) process, Ostwald process, and plasma-based process for nitrogen fixation.

In addition to ammonia, nitric acid (HNO_3_) ranks among the most widely produced chemicals globally. Large-scale production is achieved by oxidizing ammonia from the H–B process through the Ostwald process, developed by Wilhelm Ostwald.^8^ As illustrated in Figure 1, this process converts ammonia to nitric acid in two steps. First, ammonia is oxidized by oxygen over a rhodium–platinum gauze catalyst at 600–900 °C and 4–10 bar, producing nitric oxide (NO) and water. In the second stage, the NO is rapidly cooled to ∼50 °C to avoid back-reactions (i.e., decomposition to N_2_ and O_2_), then further oxidized to nitrogen dioxide (NO_2_) and absorbed in water to form HNO_3_, achieving an overall yield of ∼98%.^9^

The H–B and Ostwald processes are essential for modern agriculture and global food supply, but are highly energy-intensive, collectively consuming ∼1–2% of global energy annually.^10^ To overcome the limitations of these conventional methods, it is essential to develop nitrogen fixation technologies that are both environmentally sustainable and economically viable. Plasma-based nitrogen fixation is a promising alternative, offering low capital and operational costs through the use of compact, decentralized, modular reactors that enable instant switch on/off operation and do not require extensive infrastructure. This adaptability allows on-site, on-demand fertilizer production using only air, water and renewable electricity, making plasma electrification technology an ideal candidate for sustainable nitrogen fixation.

### 1.2 Plasma Technology

Plasma, the fourth state of matter, consists of energetic electrons and various reactive species, forming an electrically quasi-neutral and conductive medium that responds to electromagnetic fields.^11,12^ Artificial plasmas, commonly generated via electric discharges, can exist under diverse pressure and temperature conditions, and are broadly categorized into thermal and nonthermal plasmas (NTPs). Unlike thermal plasmas, NTPs are characterized by a significant temperature disparity between electrons and heavy species, enabling highly energetic electron-driven chemistry down to near-ambient gas temperatures.

These nonequilibrium features make NTPs particularly attractive for plasma-based gas conversion processes,^13–19^ including nitrogen fixation under mild conditions. High-energy electrons facilitate activation of inert N_2_ molecules via excitation, ionization, or dissociation, allowing the process to proceed without thermal input. As illustrated in Figure 2, NTP-enabled nitrogen fixation exhibits the lowest theoretical energy consumption (EC) among known processes^20^ and offers compatibility with intermittent renewable energy sources due to its rapid on/off controllability and modularity, making it flexible for decentralized ammonia and nitric acid production.

**Figure 2:**
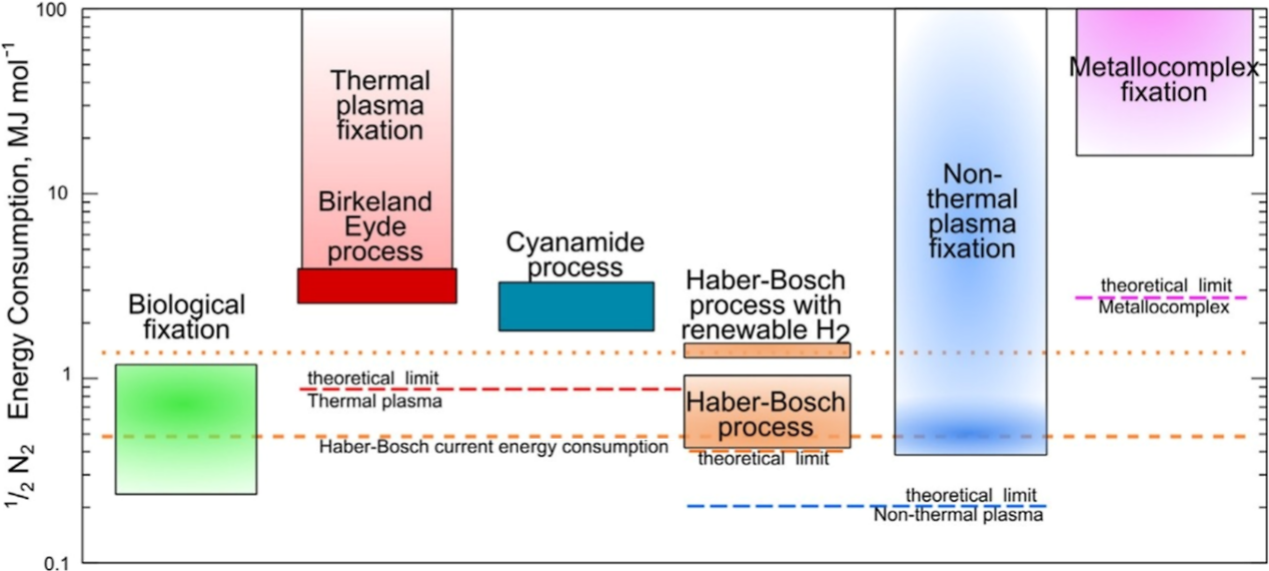
Energy consumption for different nitrogen fixation processes. Reproduced with permission from ref 20 Copyright 2015 Elsevier.

Special emphasis has been placed on nitrogen oxidation using air due to its abundance, low cost, and potential for direct NO_*x*_ generation. The formation of NO_*x*_ from N_2_ and O_2_ is an endothermic process with a relatively low standard enthalpy of 0.09 MJ mol^–1^.^21^ Thermochemical NO_*x*_ synthesis requires high temperatures and is limited by thermal equilibrium, resulting in substantial energy consumption.^22^ Theoretical studies indicate that the minimum energy required for thermochemical NO_*x*_ production exceeds 2 MJ mol^–1^, which is more than ten times higher than the energy demand for plasma-based NO_*x*_ production.^20,22^ In addition, the theoretical EC for NTP-driven nitrogen oxidation (∼0.2 MJ mol^–1^) is ∼2.5 times lower than that of the H–B process.^20^ The resulting NO_*x*_ products can be used directly in applications such as medicine^23^ or further converted into nitrates, nitrites, or ammonia. These advantages, including flexibility, low environmental impact, and process decentralization, highlight the promise of NTP-based nitrogen fixation.

However, the performance of plasma-based nitrogen fixation remains highly dependent on factors such as plasma type, reactor configuration, operating conditions, and catalyst integration. This Perspective critically evaluates recent advances in plasma-based nitrogen oxidation technologies, with particular focus on these governing variables. Section 2 discusses gas-phase NO_*x*_ production, highlighting key reaction mechanisms and the influence of operating parameters. Section 3 addresses gas–liquid interactions, such as oxynitride formation and NO_*x*_^–^ reduction to ammonia. Section 4 highlights outstanding challenges and presents a forward-looking perspective to inspire future developments in the field.

## 2 Plasma-Based Gas-phase NO_*x*_ Synthesis

Nitric oxide can be synthesized from air or N_2_–O_2_ mixtures via plasma discharge, which enables direct formation of NO and NO_2_ (collectively referred to as NO_*x*_). The nitrogen oxidation reaction R1 is

R1 $$N_{2}+O_{2}\to 2NO$$

is endothermic and typically requires high energy due to the strong N≡N bond. NTP facilitates this conversion at lower bulk gas temperatures by leveraging energetic electrons and reactive species, making it feasible under mild conditions. The following section introduces the primary types of plasma reactors employed for NO_*x*_ synthesis, highlights recent research developments, and outlines the fundamental mechanisms involved.

### 2.1 Types of Plasma Reactors for NO_*x*_ Synthesis

Numerous reactor configurations have been developed, each differing in EC and NO_*x*_ yield. Historically, the earliest attempt at plasma-driven nitrogen fixation was the Birkeland–Eyde (B–E) process, which used a thermal arc to produce NO from air. Rapid quenching to 800–1000 °C was necessary to suppress the reverse reaction. Despite various optimizations, the B–E process remained inefficient compared to the H–B process, producing only 1–2% NO at an EC of 2.4–3.5 MJ mol^–1^.^24,25^ In contrast, NTP has demonstrated reduced EC due to its nonequilibrium characteristic.^26^

Dielectric barrier discharge (DBD) is one of the most extensively studied NTPs for gas-phase reactions, including NO_*x*_ synthesis. It operates via alternating or pulsed voltage applied across electrodes, separated by at least one dielectric barrier. This configuration enables self-limiting microdischarges within the gap and prevents the transition to spark or arc discharge modes. DBD typically operates in filamentary mode at atmospheric pressure. Reactor geometries include parallel-plate and coaxial cylindrical designs, with dielectrics made of glass, ceramics, or polymers. DBD reactors are compact, scalable, and suitable for continuous-flow operation. Although initially studied for NO_*x*_ removal, recent work shows promise for NO_*x*_ generation, especially when catalysts are incorporated within the plasma zone. These variants, such as packed-bed DBD and ferroelectric DBD, enhance selectivity and energy efficiency.^17,23,27,28^

Gliding arc (GA) is a transitional plasma type that combines thermal and nonthermal characteristics, offering lower EC for NO_*x*_ synthesis at atmospheric pressure. A classical GA reactor features two diverging flat electrodes and a gas nozzle (Figure 3a). An arc ignites at the narrowest gap and is carried downstream by gas flow until extinguished, after which the cycle repeats. In GAs, electron temperatures reach 1–2 eV, enabling vibrational excitation and dissociation of N_2_ and O_2_.^29^ However, in standard flat-electrode designs, only a limited fraction of gas passes through the arc. Novel reactor designs, such as rotating GA (RGA, Figure 3b), GA plasmatron,^30^ and multielectrode configurations,^31,32^ have been proposed to increase plasma residence time and improve both conversion and energy efficiency.

**Figure 3:**
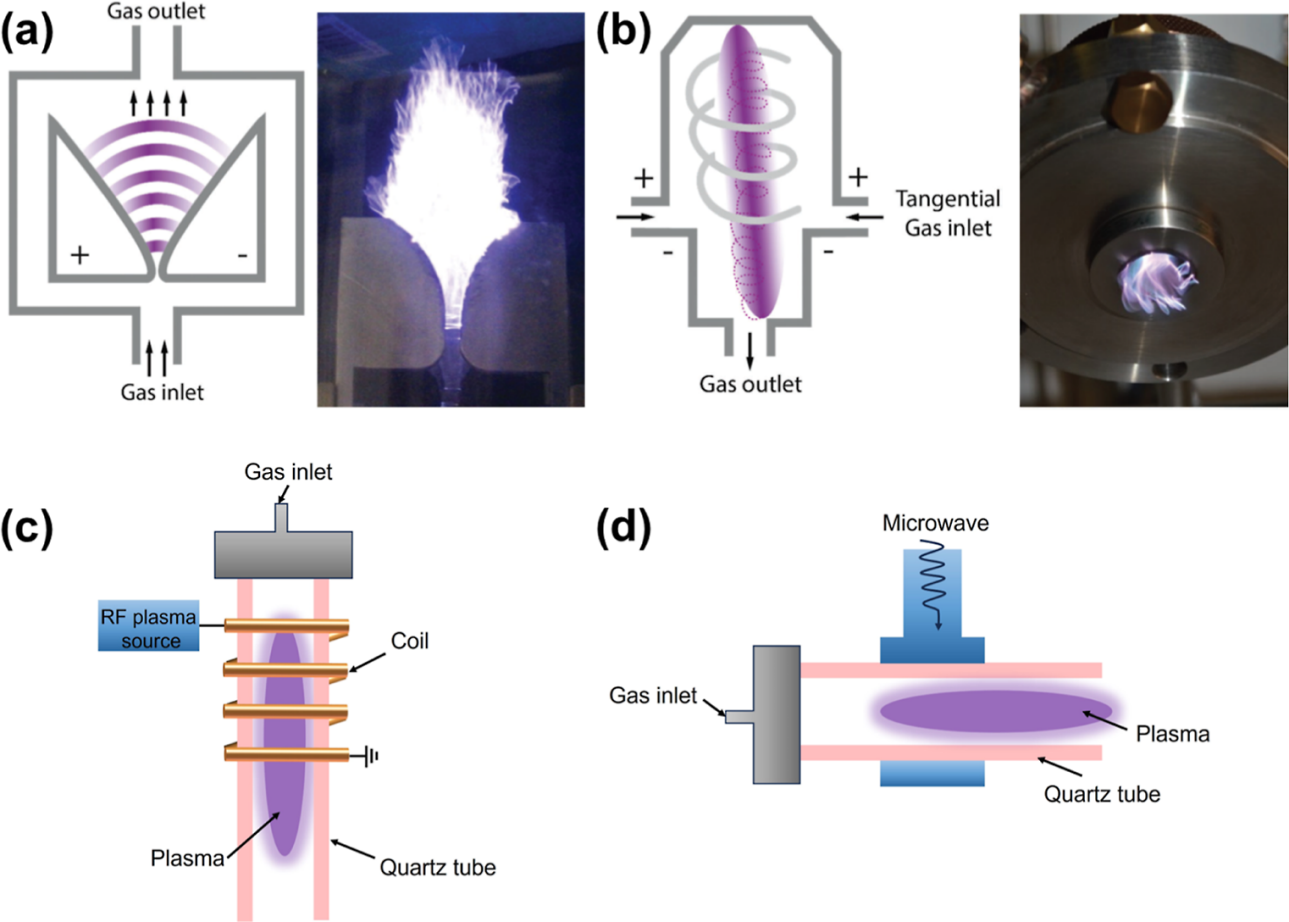
Schematic structures (top) and pictures (bottom) of (a) a classic flat GA reactor and (b) an RGA reactor. Adopted from ref 46 Schematic of (c) RF plasma, and (d) MW plasma.

Unlike DBD and GA, radio frequency (RF) and microwave (MW) plasmas are electrodeless, driven by high-frequency electromagnetic fields. This eliminates electrode degradation issues, making them suitable for harsher environments.^33^ These plasmas can operate across pressure ranges from millitorr to atmospheric levels, though lower pressures are typically used to enhance efficiency.^28^ RF plasmas (1–500 MHz, commonly 13.56 MHz) can be generated via capacitive or inductive coupling, promoting sustained plasma–field interaction (Figure 3c).^34^ MW plasmas (typically 2.45 GHz) are produced using devices such as traveling wave reactors and plasma torches (Figure 3d).^35^ In both systems, electron energies average around 1 eV due to relatively low electric fields (<50 Td), which is favorable for activation through vibrational excitation. These setups show strong potential for coupling with catalysts, improving product yield and reducing energy losses. However, their power supply systems are more complex and costly.^36^

Alternative NTP systems, such as transient spark,^32,37–40^ plasma jets,^41–43^ glow discharge,^32,44^ and corona discharge,^45^ have also been explored for NO_*x*_ synthesis. While some of these exhibit promising results at laboratory scale, their scalability and industrial relevance remain limited. Ultimately, the choice of plasma type depends on process requirements, including feed composition, operating conditions, and system integration. A detailed comparison of different plasma types is in Section 2.2, which discusses the current research progress.

### 2.2 Recent Advances in Plasma-Based NO_*x*_ Synthesis

As mentioned above, a range of plasma reactors has been explored for gas-phase NO_*x*_ synthesis. Their performance is influenced by plasma type, reactor design, and operating conditions. This section reviews recent developments, highlights key findings across different plasma systems, and identifies challenges for further advancement.

#### 2.2.1 Effect of Plasma Types on NO_*x*_ Synthesis

Figure 4 summarizes NO_*x*_ yields and EC reported across various plasma types. Notably, MW plasma with a magnetic field under reduced pressure achieved a record-low EC of 0.28 MJ mol^–1^ and a NO_*x*_ concentration of 140,000 ppm.^47^ However, these results have not been replicated, and the additional energy required for vacuum systems often offsets the gains.

**Figure 4:**
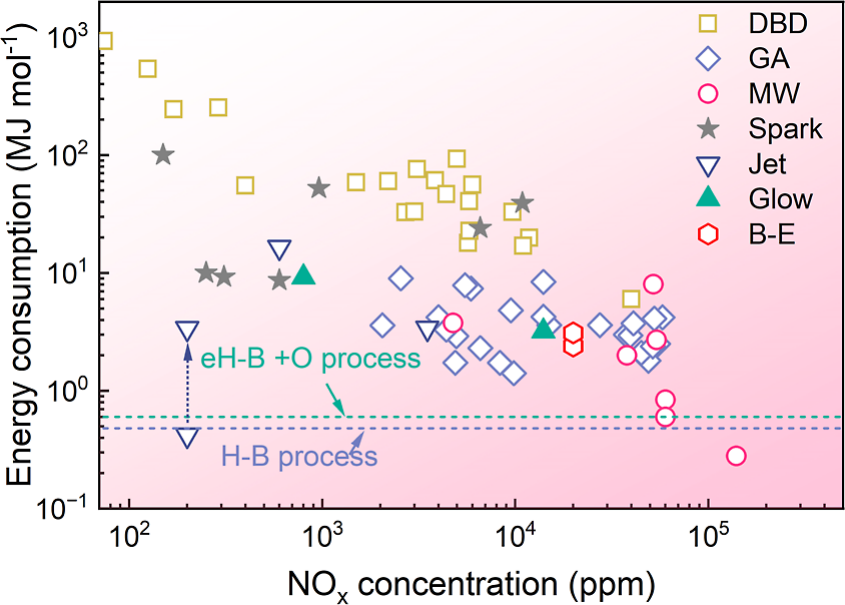
Energy consumption and concentration of nitric oxides from plasma synthesis reported in the literature. Reference: DBD,^32,48–53^ GA,^22,30,32,54–60^ MW discharge,^47,52,61–64^ spark discharge,^37–39,65^ plasma jet,^41,43,66^ and glow discharge.^67,68^

DBD reactors, though simple and scalable, typically yield high ECs for NO*_x_* synthesis. Integration with catalysts or nanosecond-pulsed power has improved performance.^69^ For example, Cheng et al.^70^ demonstrated that nanosecond-pulsed DBD enhances metastable nitrogen formation, while Tang et al.^53^ observed N_2_O_5_ production via NO_3_ and NO_2_ interactions in a DBD plasma. Dielectric materials^71^ and magnetic fields^72^ have also shown measurable impact on NO/NO_2_ ratios and EC.

GA reactors have demonstrated superior characteristics in balancing EC and NO_*x*_ yield. Variations such as propeller GA and RGA increase discharge residence time and conversion.^30,32,55,56,73^ For instance, Patil et al.^59^ reported 1.0% NO_*x*_ at an EC of 1.4 MJ mol^–1^ using a milli-scale GA reactor. Incorporating a TiO_2_ catalyst, Lei et al. further reduced the EC by 40%.^74^

MW and RF plasmas generally require higher input power than other plasma types due to wave transmission losses. At atmospheric pressure, Kim et al.^52^ reported an MW system achieving a minimum EC of ∼3.8 MJ mol^–1^. Better performance is typically observed under reduced pressure; for example, Bahnamiri et al.^63^ achieved a 7% NO_*x*_ yield at an EC of 8 MJ mol^–1^ using a pulsed low-pressure MW plasma system. The lowest reported EC (0.28 MJ mol^–1^) was obtained by Asisov et al.^47^ using a magnetically enhanced MW plasma, with over 90% plasma energy absorption. However, this result relied on highly specific conditions, including sub-100 Torr pressure, cryogenic cooling, and electron-cyclotron resonance, which pose significant challenges for practical deployment. Energy-efficient performance in RF plasmas has been reported less frequently in the last century.^75–77^

Spark discharges offer high reactivity but require significantly higher EC (5–40 MJ mol^–1^),^38–40,45,65^ which is generally 1–2 orders of magnitude higher than that of GA. Janda et al.^38^ and Pei et al.^32^ demonstrated performance improvements by modifying electrode geometry and employing pulsed power. However, product selectivity and EC vary considerably depending on configuration and frequency. The shape and positioning of electrodes, as well as the type of power supply (AC or pulse), significantly influence the characteristics of the spark discharge, thereby affecting the concentration and ratio of the resulting products.

Jet plasmas have achieved the lowest EC (0.42 MJ mol^–1^) at atmospheric pressure,^41^ though this value excluded interpulse EC. In this setup, a plasma arc is generated between the end of the pin electrode and the metal nozzle (Figure 5a), driven by the gas flow to form the jet. The discharge mechanism is similar to a low-current spark operating in pulsed mode, and the minimum EC of 0.42 MJ mol^–1^ is achieved at a flow rate of 1.5 L min^–1^ (Figure 5b), approaching the theoretical minimum EC of NTP (0.2 MJ mol^–1^). This is attributed to pulsed heating and the strong vibrational–translational nonequilibrium that promotes the nonthermal Zeldovich mechanism. However, the calculations excluded interpulse power consumption. ICCD images confirmed plasma presence during the interpulse phase, indicating that the EC may have been underestimated. When total power is considered, the corrected EC increases to 3.4 MJ mol^–1^, bringing it closer to values reported in other plasma systems.

**Figure 5:**
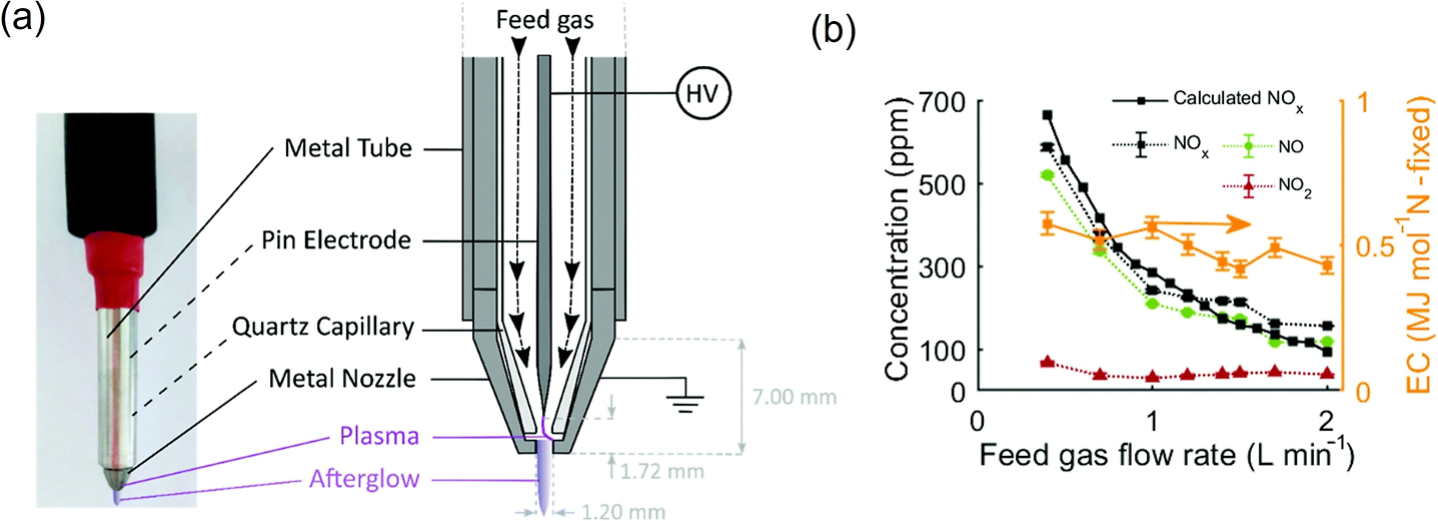
(a) Schematic of the jet, and (b) measured NO_*x*_ concentration, calculated total NO_*x*_ concentration, and measured EC. Reproduced with permission from ref 41 Copyright 2022 Royal Society of Chemistry.

Other plasma systems, such as corona discharge, streamer discharge, and transient spark, have been explored but generally show higher EC and limited industrial relevance.^45,78–80^

#### 2.2.2 Effect of Operating Parameters

Operating parameters significantly affect NO_*x*_ yield and EC. These include electrical inputs (e.g., input power, frequency, and pulse width) and process conditions (e.g., gas flow rate, feed gas composition, pressure, and temperature).

##### 2.2.2.1 Effect of Electric Parameters

Input power directly influences plasma characteristics. Higher power generates more energetic electrons and reactive species, increasing N_2_–O_2_ collisions. This promotes bond dissociation and subsequent recombination, producing higher concentrations of NO and NO_2_. Rousseau et al.^81^ found that NO_*x*_ concentration depends on the average power, and can be scaled logarithmically with input power in a MW discharge reactor with a catalyst. A power saturation effect is widely reported, where increasing power leads to diminishing NO_*x*_ concentration gains until stabilization, inherently resulting in higher EC. Meanwhile, the effect of changing power frequency varies across different plasma systems, influencing discharge behavior and NO/NO_2_ ratios. Patil et al.^59^ demonstrated a 31% increase in NO_*x*_ concentration when raising the frequency from 7 kHz to 9 kHz in a mini-scale GA reactor. However, this increase in frequency also increased the specific energy input (SEI) and altered discharge properties. Despite the beneficial effect of increasing frequency on NO_*x*_ concentration, operating the reactor at a lower frequency of 7 kHz was more energy efficient.^59^ However, the impact of frequency on NO_*x*_ production varies across systems. For instance, in the pulsed MW plasma, average power remains the dominant factor;^82^ in the spark discharge, the EC remains largely constantly with increasing frequency.^38^

Variations in both power and frequency can trigger transitions in arc discharge modes^56,59^ often reflected in changes to voltage and current waveforms, potentially leading to either performance gains or losses. Pulse width also plays a role, particularly in pulse-driven plasma reactors. At a fixed frequency, increasing pulse width enhances power deposition and can reduce EC. Pei et al.^32^ showed that in pin-to-plane spark discharges, increasing the pulse width from 100 to 260 ns under three voltage conditions lowered the EC for NO_*x*_ production. They identified the gas breakdown phase as the most energy-intensive, suggesting that improving breakdown efficiency could enhance overall energy efficiency. However, in pulsed MW discharges operated in air, NO_*x*_ production is largely insensitive to pulse duration or frequency, with average power being the dominant factor.^81^ Additionally, discharge current affects NO_*x*_ production, though its impact varies across discharge types. In propeller arc discharges, higher currents reduced EC,^32^ whereas in DC glow discharges, the lowest EC consistently occurred at ∼40 mA, regardless of flow rate. Furthermore, beyond this current, EC could increase.^44^

##### 2.2.2.2 Effect of Process Parameters

Reduced flow rates increase residence time and NO_*x*_ yield but can result in higher SEI. At a fixed power, increasing the feed gas flow rate shortens the residence time, reducing the interaction between plasma and reactants. Conversely, decreasing the feed gas flow rate enhances SEI, promoting higher NO_*x*_ concentrations at lower inlet gas flow rates. Specifically, NO_2_ selectivity decreases with increased flow rate due to insufficient time for NO to oxidize to NO_2_. Studies using MW and GA reactors confirm the trade-off between NO_*x*_ concentration and EC.^64,82^ In RGA reactors, reversing vortex flow improves residence time and heat insulation.^46^

Feed gas composition also influences the performance of plasma-based NO_*x*_ production. Optimal N_2_/O_2_ ratios (typically 30–50% O_2_) vary with reactor type.^10,22,82,83^ Pei et al.^32^ reported maximum yield and energy efficiency at 33.3% O_2_. Notably, using air increases EC by only 16%, a practical and readily available feedstock. Furthermore, adding Ar can enhance plasma uniformity (e.g., in a DBD) but may reduce NO_*x*_ yield in GA due to flow effects and reduced air concentration.^54,84^ H_2_O addition (up to ∼12%) can promote NO formation by generating OH and NH radicals, but excess H_2_O decreases energy efficiency.^31,74^ Modeling studies also suggest that O_3_ addition may suppress reverse Zeldovich reactions by converting NO to NO_2_,^30^ though more experimental validation is required.

Reaction temperature effects are complex and plasma-type dependent. For DBD, increasing temperature shifts the discharge mode from O_3_-mode to NO_*x*_-dominated, improving energy efficiency for NO_*x*_ synthesis.^85,86^ For RGA, fast quenching prevents NO decomposition at >1000 K, as reported by Majeed et al.,^57^ which could achieve the highest NO_*x*_ concentrations of ∼5.2% and lowest EC of ∼2.4 MJ mol^–1^. To describe the relationship between temperature and performance, Pei et al. defined a dimensionless factor χ that depends only on the electric field and average plasma discharge temperature to evaluate energy efficiency between different discharges.^32^

Unlike the high pressures required for the H–B process, plasma systems operate effectively at atmospheric pressure, though elevated-pressure systems have shown improved performance. The recently published low-temperature plasma roadmap emphasizes the significance of plasmas operating above atmospheric pressure.^87^ Fundamental phenomena of high-pressure plasmas in various gases, including high-pressure arc discharges, have been extensively studied both experimentally and theoretically.^88–90^ In RGA reactors, increased pressure enhances NO_*x*_ concentration, yield, and NO_2_ selectivity due to equilibrium shifts and reaction pathway optimization.^58^ Figure 6a shows that NO concentration declines beyond the optimal temperature but remains higher at elevated pressures. This is attributed to increased equilibrium NO levels and enhanced oxidation of NO to NO_2_ (P2, Figure 6b), which suppresses its recombination with O (P1, Figure 6b). Higher pressures also improve NO_2_ selectivity, supporting nitrogen fixation. Under optimal conditions (N_2_/O_2_ = 1:1, 12 L min^–1^, 3 bar), 4.9 vol % NO_*x*_ was achieved at 1.8 MJ mol^–1^ with 95.5% NO_2_ selectivity.^58^

**Figure 6:**
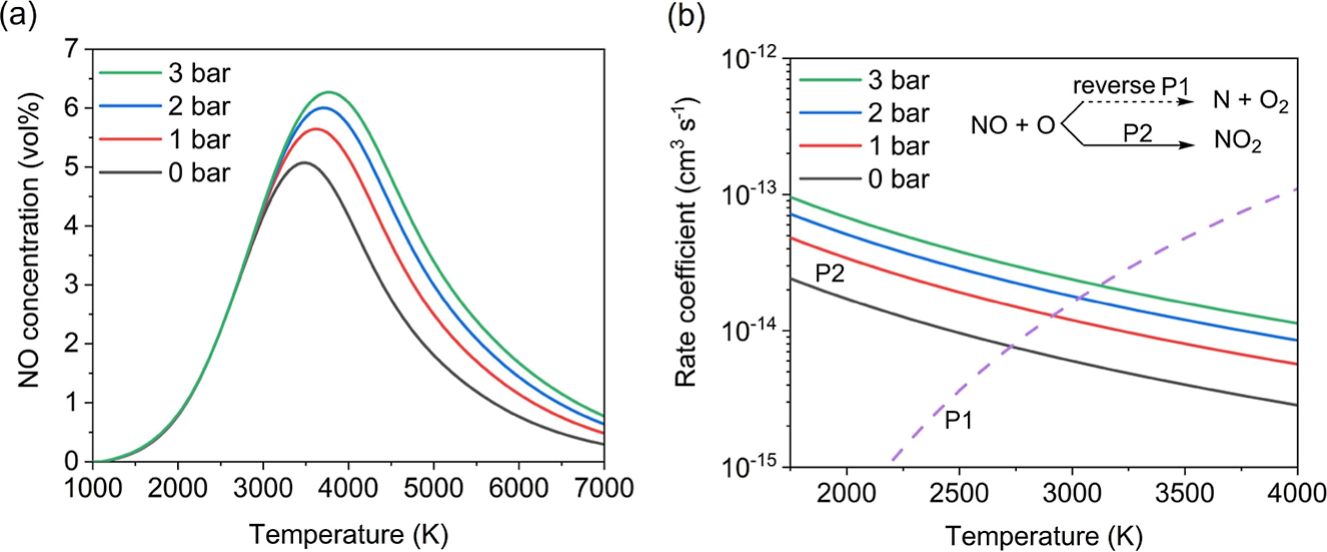
(a) Equilibrium concentration of NO as a function of temperature at different pressures (0–3 bar), and (b) rate coefficients of the pressure-independent back reaction of P1 and the pressure-dependent forward reaction of P2 as a function of temperature. Reproduced with permission from ref 58 Copyright 2023 American Chemical Society.

In summary, the NO/NO_2_ ratio can be tuned by adjusting electrical and process parameters. However, selectivity and EC may still vary due to differences in reactor design, and the presence of catalysts can significantly alter performance.

#### 2.2.3 Plasma-Catalyst Coupling

Catalysts can influence plasma reactions both physically and chemically. They modify discharge characteristics such as discharge regime, electron density, temperature, and electric field distribution. Additionally, active sites on the catalyst surface introduce highly reactive species that alter reaction pathways. Catalysts can be placed directly in the discharge gaps, coated on electrode surfaces within the plasma region, or positioned outside the discharge region to avoid unwanted changes in discharge properties and instability caused by direct plasma-catalyst interactions. Figure 7 illustrates different configurations of plasma-catalytic systems for NO_*x*_ production across various plasma types. In Figure 7a, the catalyst bed is situated within the DBD plasma field, allowing close contact between plasma and catalysts, known as in-plasma catalysis. This configuration is widely used in plasma catalysis, including plasma-catalytic NO_*x*_ production. The synergistic effect in this setup is significant and complex, as the physicochemical properties of both plasma and catalyst mutually influence and modify each other.

**Figure 7:**
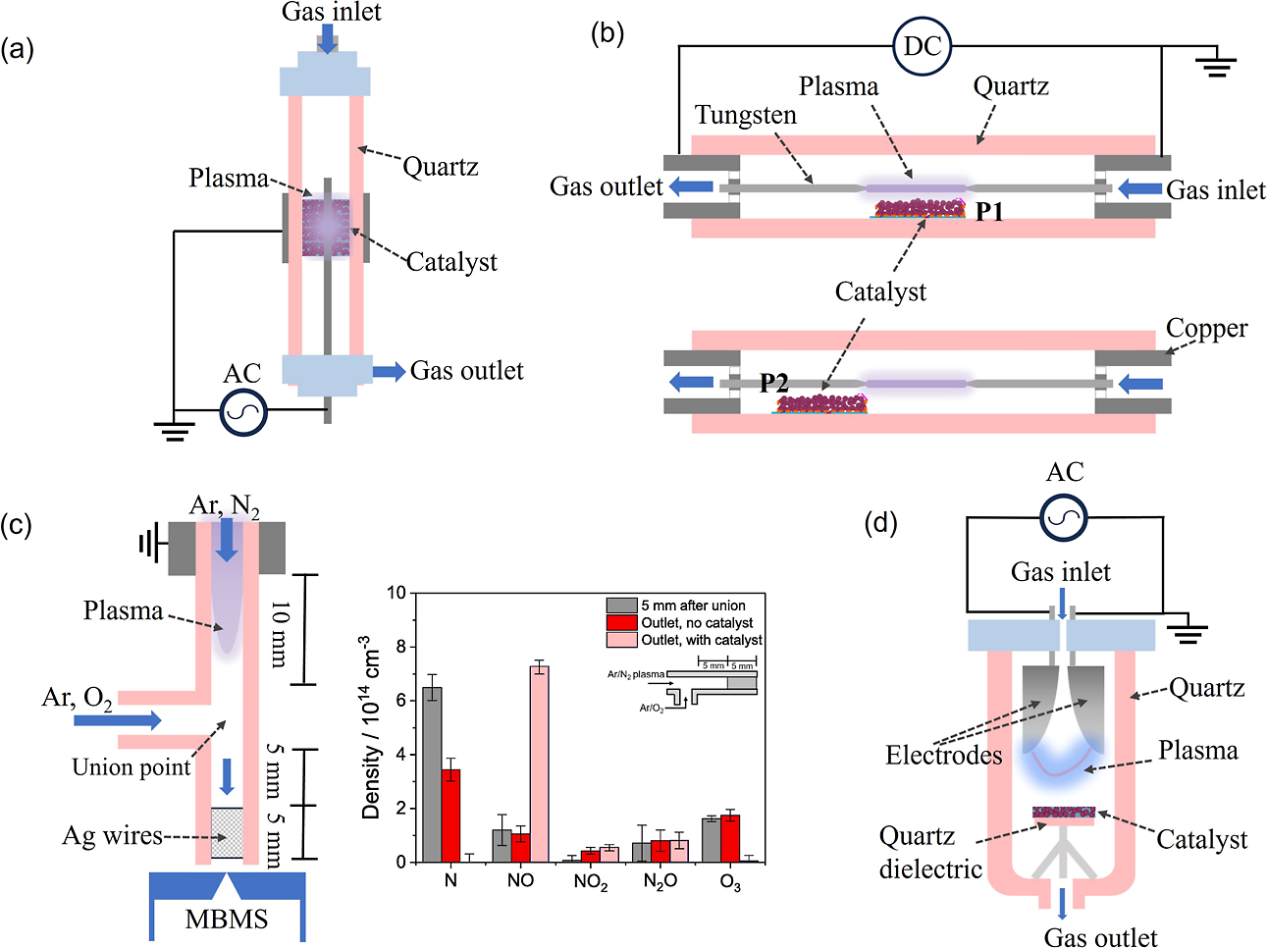
Schematic of the plasma-catalytic NO_*x*_ production in the (a) DBD reactor, (b) glow discharge reactor,^44^ (c) plasma jet,^93^ and (d) GA reactor.^74^

Remarkable improvements in NO_*x*_ yield have been reported using the in-plasma catalysis configuration. Sun et al.^91^ demonstrated improved NO_*x*_ yield with Cu-ZSM-5 catalysts at >623 K. Patil et al.^49^ and Ma et al.^50^ reported yield enhancements with γ-Al_2_O_3_ and BaTiO_3_, although EC remained high (18–20 MJ mol^–1^), where NO_2_ was the predominant product. Rouwenhorst et al.^92^ demonstrated that employing MgO as a sorbent increased the NO_*x*_ yield in a DBD reactor by a factor of 15. The in situ removal of NO_*x*_ species from the plasma reaction likely prevents their subsequent decomposition by the plasma, thereby reducing EC. Despite these advancements, the EC achieved with this configuration remains significantly higher than state-of-the-art results, primarily due to the inherent limitations of the DBD reactor.

To reduce the EC of plasma-catalytic NO_*x*_ production, alternative plasma types and reactor configurations have been explored. In these configurations, catalysts are positioned adjacent to, but not directly within, the discharge area (several mm away), as shown in Figure 7b–d. In glow discharge systems, positioning Al_2_O_3_ below the discharge zone enhanced yield, but the effect vanished when the catalyst was moved further away,^44^ indicating the importance of short-lived intermediates. Recently, Bayer et al.^93^ investigated the influence of surface reactions, gas-phase reactions, and mass transport on plasma-catalytic NO formation using a DBD-based plasma jet. The system configuration (Figure 7c) involves feeding O_2_ into the afterglow and placing an Ag wire catalyst downstream of the plasma jet, 5 mm away from the union point. This setup allows quantification of species density via molecular beam mass spectrometry (MBMS), enabling detailed analysis of plasma-assisted N_2_–O_2_ reaction pathways and time scales by measuring the consumption of plasma-derived N and the formation of NO in both the gas phase and on catalyst surfaces. As depicted in Figure 7c, gas-phase reactions consume N but do not selectively form NO. The presence of the nonporous Ag wire catalyst enhances N conversion to NO, primarily through mass transfer processes rather than surface reactions. However, when NO density exceeds a threshold, N cannot diffuse to the catalyst surface faster than it is consumed in the gas phase by reactions with NO, limiting the effectiveness of heterogeneous catalysts to scenarios where diffusive transport of N to the catalyst surface outpaces gas-phase consumption by reactions with NO.

GA-based systems have demonstrated promising energy-efficient NO_*x*_ production, outperforming other types of plasma. However, incorporating catalysts into GA reactors presents challenges due to their distinctive discharge characteristics. Meng et al.^94^ used TiO_2_-coated quartz to create a dielectric-boosted GA reactor, improving yield by 23% and reducing EC by 82% (Figure 7d). Lei^74^ applied nano-TiO_2_ in an RGA reactor and achieved a 40% EC reduction, attributed to oxygen vacancies and electron–hole pair formation that enhance O_2_ dissociative adsorption.

These studies indicate that decoupling parameters in complex plasma systems remains challenging, as plasma–surface and gas-phase interactions are poorly understood. Mechanistic insight beyond empirical optimization is essential. Rather than isolating performance factors, a deeper understanding of the underlying processes can more effectively guide the optimization of plasma-based NO_*x*_ production.

### 2.3 Mechanisms of Plasma-Based NO_*x*_ Synthesis

Understanding the reaction mechanisms and identifying key intermediates are crucial for optimizing plasma-assisted NO_*x*_ synthesis. Mechanistic insights guide reactor design, operating conditions, and catalyst development, enabling more efficient and sustainable nitrogen fixation. This section provides an overview of the main gas-phase pathways (Section 2.3.1) and plasma–catalyst interactions (Section 2.3.2).

#### 2.3.1 NO_*x*_ Synthesis without a Catalyst

Plasma consists of electrons, ions, radicals, and excited species. Figure 8a illustrates the typical time scales of elementary processes in a plasma region. Kim et al.^95^ identified two distinct stages in gas-phase plasma reactions based on the streamer propagation time scale: a primary and a secondary process. The primary stage, occurring on the nanosecond scale, involves electron-driven processes, such as ionization, excitation, dissociation, charge transfer, and vibrational–vibrational (VV) and vibrational–translational (VT) relaxations, which are strongly influenced by the power source and reactor design. The secondary stage comprises slower chemical reactions, including radical–radical and radical–neutral reactions, with neutral–neutral reactions occurring on millisecond or longer time scales, and playing a critical role in determining the overall plasma performance. Electron impact reactions are crucial in transferring energy to the system, initiating the dissociation of N_2_ and O_2_ molecules.^30,37^ Figure 8b summarizes the possible fractions of electron energy loss through different channels under various reduced electric fields (E/N) in N_2_/O_2_ = 80/20 gas mixtures at atmospheric pressure.^22^ At low E/N values (<30 Td), most of the electron energy is transferred to vibrational excitation of O_2_ and N_2_. As E/N increases (30–200 Td), ionization and dissociation of both species gradually become important and cannot be neglected. At high E/N (>200 Td), ionization dominates, while rotational and vibrational excitations contribute negligibly, demonstrating the strong dependence of the electron energy distribution on the reduced electric field.

**Figure 8:**
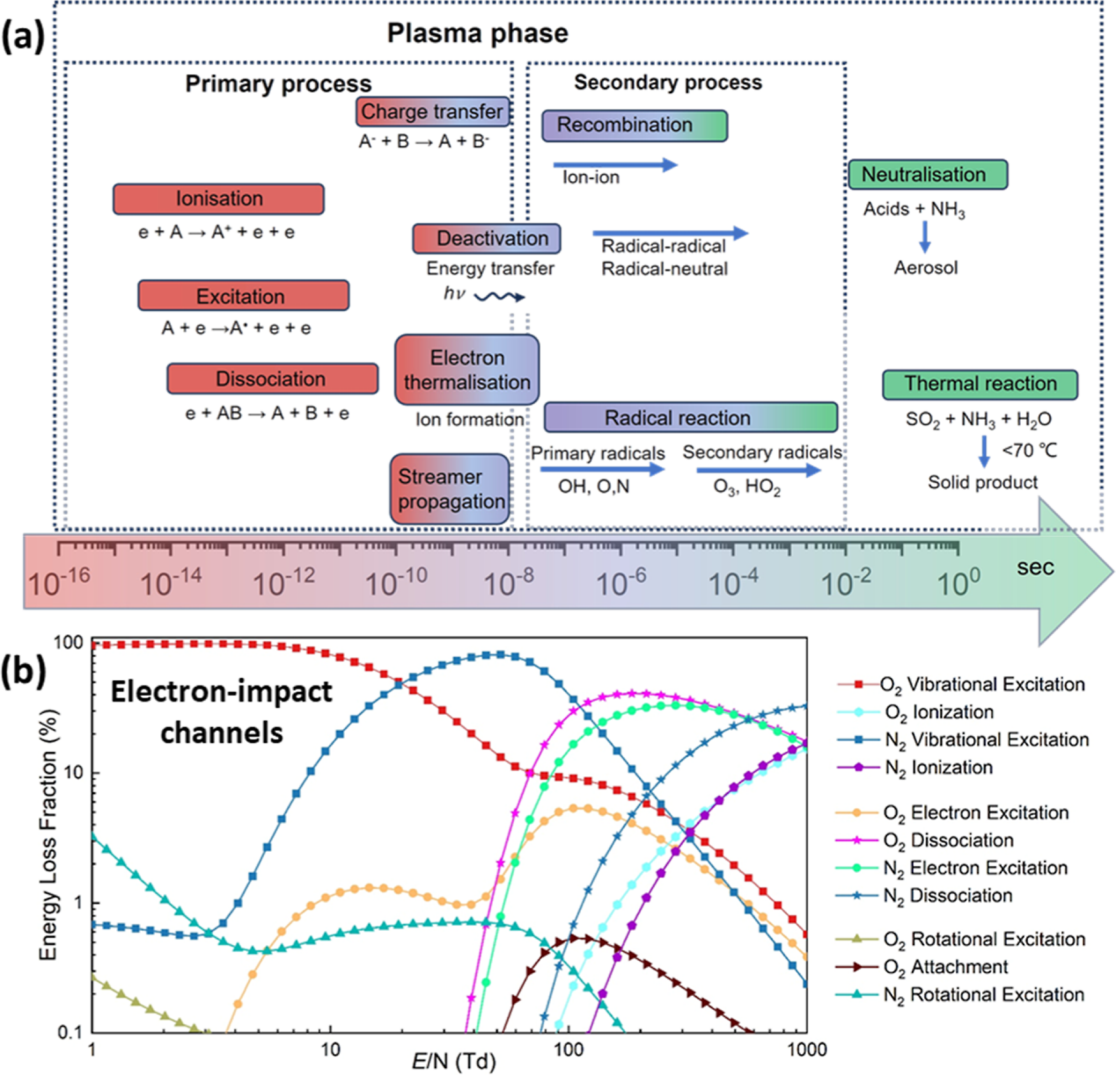
(a) Time scale of different reaction types within a plasma regime. Adapted with permission from ref 96 Copyright 2004 Wiley-VCH GmbH. (b) Energy loss fraction of different channels of N_2_ and O_2_ as a function of E/N. The results are calculated under N_2_/O_2_ = 80/20 at atmospheric pressure.

The direct dissociation of N_2_ (R2 and R3) requires high threshold energies (11–13 eV), making it inefficient.

R2 $${e^{-}+N_{2}\to e^{-}+N+N,\varepsilon }_{th}=11\;eV$$

R3 $$e^{-}+N_{2}\to e^{-}+N+N\left(2D\right),\varepsilon_{th}=13\;eV$$

In NTPs, vibrational and electronic excitations (R4 and R5) provide more energy-efficient alternatives^11,22^

R4 $$e^{-}+N_{2}\to e^{-}+N_{2}\left(v1\right),\varepsilon_{th}=0.28\;eV$$

R5 $$e^{-}+N_{2}\to e^{-}+N_{2}\left(A3\right),\varepsilon_{th}=6.17\;\mathrm{eV}$$

The dominant electron reaction in plasma discharges can be the vibrational excitation of N_2_ and O_2_, particularly when the reduced electric field is below 100 Td. The energy efficiency for NO_*x*_ formation is determined by the method used to break the strong (∼10 eV) bond of the N_2_ molecule. As noted earlier, DBD is normally operated at 100–200 Td, where vibrational excitation is less pronounced. Under these conditions, direct dissociation, which requires high electron energies, becomes the primary pathway within the reacting system. Indeed, previous studies^11^ have shown that the energy efficiency of DBD-enabled NO_*x*_ production is limited to ∼3%, owing to the very high dissociation threshold of N_2_. This limitation explains the higher EC or lower energy efficiency observed in DBD-based NO_*x*_ synthesis.

These excited species, particularly vibrationally excited N_2_, act as energy reservoirs and dominate NO formation in pulsed spark and GA discharges.^30,37^ Modeling studies show that more than 98% of NO in GA arises from N_2_(*v*),^30^ highlighting the critical role of vibrational excitation of N_2_. O atoms then react with N_2_(*v*) to produce NO via the Zeldovich mechanism

R6 $$O+N_{2}\left(v\right)\to NO+N$$

This is followed by

R7 $$N+O_{2}\left(g,v\right)\to NO+O$$

Meanwhile, vibrational-translation relaxations, which contribute to gas heating, influence both gas temperature and the reaction balance.^97^ Vibrationally excited N_2_ molecules can either collide directly with free electrons, undergoing further excitation and de-excitation processes, or react with other neutral molecules via VV exchanges.^98^ For instance, N_2_–O_2_ VV collisions transfer energy from N_2_ to O_2_, leading to de-excitation of vibrational N_2_ levels and thereby limiting NO formation.^30^

Oxygen atoms produced in the system can react with vibrationally excited N_2_ molecules, thereby completing the reaction cycle described in reaction R6. As a result, both nitrogen and oxygen atoms are simultaneously consumed and regenerated through these processes. Reactions R6 and R7, collectively known as the Zeldovich mechanism, constitute the most energy-efficient pathway for NO_*x*_ formation. This mechanism is initiated by atomic nitrogen and oxygen, which are generated via dissociation reactions. Vervloessem et al.^30^ demonstrated that in GA, N atoms are mainly produced through direct electron-impact dissociation of N_2_ (ground or vibrationally excited states), establishing a direct correlation between N atom production and the N_2_ fraction. In contrast, atomic oxygen is generated via two primary pathways: direct electron-impact dissociation of O_2_ (in ground or vibrationally excited states), and dissociation induced by collisions with electronically excited N_2_ molecules. The contribution of each mechanism depends on the feed gas composition, although the overall atomic oxygen density is relatively insensitive to feed gas variations. Among these pathways, reaction R6 is widely recognized as the rate-limiting step. Notably, nitric oxide can also be produced through various ion–molecule reaction channels

R8 $$O_{2}^{+}+N_{2}\to {NO}^{+}+N$$

R9 $$N_{2}^{+}{+O}_{2}\to {NO}^{+}+NO$$

The rate coefficients of these alternative pathways are substantially lower than those of reactions R6 and R7, indicating that the latter remain the dominant contributors to NO formation. The subsequent oxidation of NO to NO_2_ is strongly influenced by both oxygen concentration and system temperature.

Minimizing NO_*x*_ loss processes is critical for enhancing energy efficiency. Vervloessem et al.^30^ evaluated the impact of various NO_*x*_ loss reactions in GA, as shown in Figure 9a and b. The main NO loss pathway involves reactions of NO with O atoms or molecules to form NO_2_. While NO_2_ can be reduced back to NO, this does not represent a net NO_*x*_ loss. Reactions in which NO reacts with O or N atoms to form N + O_2_ or O + N_2_ (the reverse Zeldovich mechanism) occur less frequently but are crucial, as they inhibit NO formation and effectively terminate the reaction chain. Simulations suggest that excluding these reactions from the model could increase NO_*x*_ yield by an order of magnitude. Consequently, suppressing these pathways, for instance through rapid quenching or in situ NO extraction, may enhance NO production efficiency.

**Figure 9:**
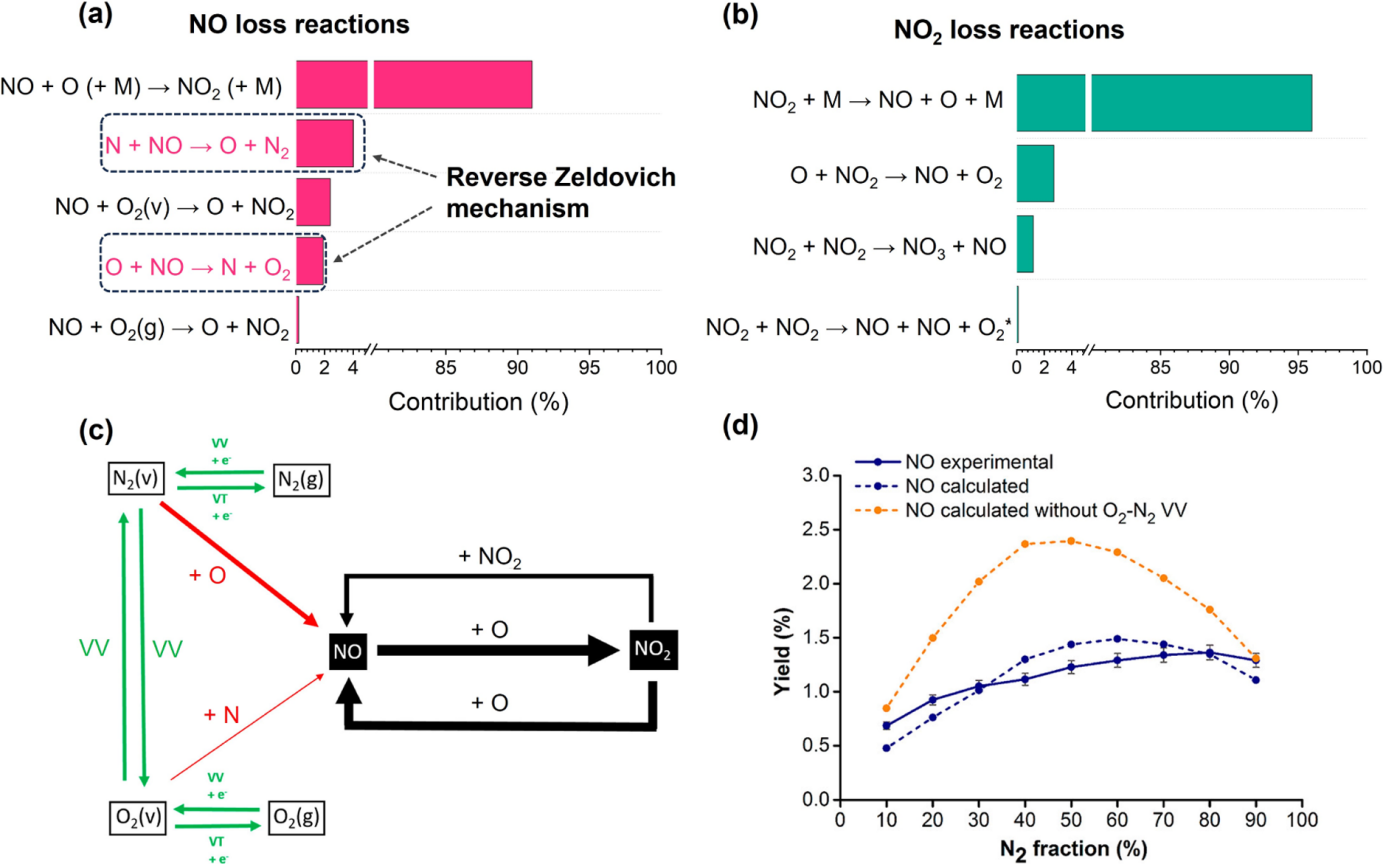
Dominant loss reactions of (a) NO and (b) NO_2_, (c) reaction scheme of the main reaction paths for NO_*x*_ synthesis (the thickness of the arrow lines corresponds to the importance of the reactions The two steps of the Zeldovich mechanism are depicted in red; the VV and VT exchanges and electron impact reactions are in Green), and (d) experimental and calculated yield of NO (blue) compared to the calculated yield of NO without including N_2_–O_2_ VV exchanges in the chemistry set (orange), as a function of N_2_ fraction in the feed gas. Reproduced with permission from ref 30 Copyright 2020 American Chemical Society.

Beyond chemical loss mechanisms, vibrational kinetics also limit NO_*x*_ formation. Among known pathways, vibrational-induced dissociation is the most energy-efficient, as low-energy electrons preferentially excite lower vibrational levels of N_2_ via electron impact. These levels are progressively populated through VV energy exchange. However, as shown in Figure 9c, VV exchanges occur not only among identical molecules (N_2_–N_2_, O_2_–O_2_), but also between vibrationally excited N_2_ and O_2_.^30^ Given the rapid depopulation of O_2_ vibrational states, N_2_–O_2_ VV interactions reduce N_2_ vibrational energy, thereby hindering dissociation. Simulations (Figure 9d) show higher NO_*x*_ yields when these interactions are omitted. Although controling such exchanges is challenging, preheating the feed gas may promote thermal O_2_ dissociation and vibrational excitation. Further research is required to evaluate the practical viability of this approach.

Although vibrationally induced dissociation is energetically favorable, modeling remains challenging due to the lack of accurate cross-section and rate data for vibrationally and electronically excited species. Current approaches rely on approximations (e.g., Fridman–Macheret α-model^11^) and empirical correlations.^99–101^ A combination of kinetic modeling and advanced diagnostics is therefore required to elucidate the roles of key intermediates and enhance mechanistic understanding.

#### 2.3.2 Plasma-Catalytic Synergistic Effect

Plasma catalysis involves complex interactions between plasma species and catalytic surfaces.^87,102^ Catalysts can modify discharge characteristics by affecting electric field distribution, electron density, and microdischarge behavior.^103–105^ Figure 10a summarizes key pathways and species in plasma-catalytic NO_*x*_ synthesis.

**Figure 10:**
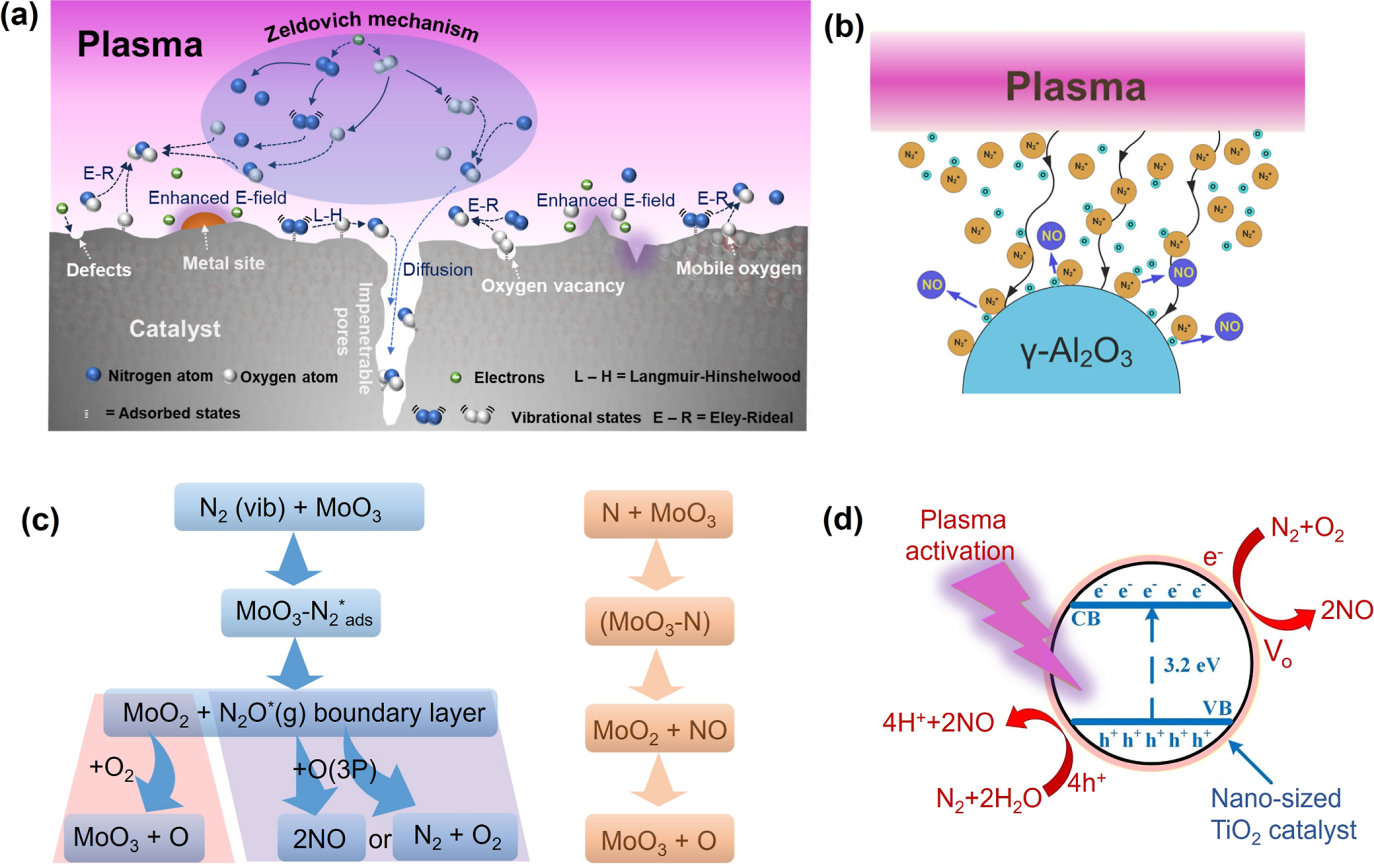
(a) Schematic of the key mechanisms and species in plasma-catalytic NO_*x*_ production. Proposed mechanisms for plasma-catalytic NO_*x*_ production in (b) DC plasma jet with γ-Al_2_O_3_ as the catalyst. Reproduced with permission from ref 106 Copyright 2023 Wiley-VCH GmbH., (c) MW plasma with MoO_3_ as the catalyst, and (d) GA with TiO_2_ as the catalyst. Reproduced with permission from ref 74 Copyright 2021 IOP Publishing.

In packed-bed DBD reactors, the curvature and roughness of catalysts enhance local electric fields, promoting microdischarges. Patil et al.^49^ found that quartz wool enhanced NO formation due to its fibrous structure with sharp edges, facilitating the formation of stronger microdischarges. Even noncatalytic supports like γ-Al_2_O_3_ can boost NO_2_ selectivity through surface reactions with NO and adsorbed oxygen under atmospheric pressure air plasma.^106^ As illustrated in Figure 10b, reactive plasma species (e.g., N_2_*, O) diffuse to alumina surfaces, where reactions such as N_2_* + O → NO + N occur more readily.^106^ These surface-adsorbed intermediates increase NO production by enhancing both proximity and lifetime.

However, the effect of catalysts on NO_*x*_ production varies across different plasma systems. For instance, in DBD-assisted NO_*x*_ production, loading various metal oxides on γ-Al_2_O_3_ had only a minor (<10%) effect on NO_*x*_ yield.^49^ Notably, WO_3_/γ-Al_2_O_3_ was the most active, while well-known oxygen activation catalysts like PbO/γ-Al_2_O_3_ or Co_3_O_4_/γ-Al_2_O_3_ showed improvements below 5%. This suggests that the primary role of catalysts in DBD-assisted NO_*x*_ production is to facilitate microdischarge formation rather than chemically interacting with plasma-activated species. In low-pressure MW reactors, Gicquiel et al.^108^ observed that MoO_3_ deposition on reactor walls significantly increased NO production, even in N_2_-only plasmas. The reduction of MoO_3_ to MoO_2_ suggested a reaction involving vibrational N_2_ and lattice oxygen, consistent with an Eley–Rideal (E–R) mechanism (Figure 10c).

TiO_2_, a widely used photocatalyst, has also been integrated into GA systems. The photocatalytic mechanism of TiO_2_, particularly in the anatase phase, involves activation by photon energy exceeding its bandgap energy of 3.2 eV. This activation generates electron–hole (e^–^–h^+^) pairs (R10) between the conduction and valence bands^109^

R10 $$TiO_{2}+hv\to e^{-}+h^{+}$$

In plasma environments, TiO_2_ is activated by electron impact rather than UV radiation.^107,110–113^ High-energy electrons generate electron–hole (e^–^–h^+^) pairs and oxygen vacancies (V_o_), enabling O_2_ adsorption and superoxide (O_2_^–^) formation (R11 and R12)­

R11 $$TiO_{2}+e^{-}\left(>3.2\;eV\right)\to e^{-}+h^{+}$$

R12 $$O_{2}+e^{-}\left(V_{o}\right)\to O_{2}^{-}$$

The activated TiO_2_ surface, with oxygen defects, may facilitate the conduction band mechanism, enhancing NO_*x*_ production (Figure 10d). Adsorbed O_2_ at V_o_ sites forms superoxide (O_2_^–^), which then react with N_2_ to produce NO. Lei et al.^74^ attributed a 40% reduction in EC to this mechanism.

Despite these promising findings, the detailed mechanisms remain poorly understood. The dominant plasma-activated N_2_ species, the nature of active catalytic sites, and the relative roles of Langmuir–Hinshelwood (L–H) vs E–R pathways are still under investigation. Addressing these gaps requires in situ diagnostics such as in situ Fourier transform infrared spectroscopy (FTIR) and in situ X-ray photoelectron spectroscopy (XPS), along with systematic studies of metal–support combinations and plasma-catalyst configurations, to gain deeper insights into the molecular-level plasma-catalytic interactions. Furthermore, the combination of GA with catalysts has rarely been investigated.

## 3 Plasma-Based Nitrogen Fixation in Liquid Phase

Beyond gas-phase oxynitride synthesis, plasma-assisted nitrogen fixation in liquids has attracted significant interest due to its unique advantages. This approach enables compact, catalyst-free systems capable of on-site, single-step fertilizer production powered by distributed renewable energy sources (e.g., wind and solar). The following section briefly discusses recent advances in plasma-based synthesis of nitrogen compounds in liquids.

### 3.1 Plasma-Based NO_*x*_^–^ Production in Liquids

Among the various nitrogen compounds, nitrate (NO_3_^–^) and nitrite (NO_2_^–^) are the predominant species in agricultural soils.^114^ Therefore, directly converting nitrogen into NO_*x*_^–^ compounds (NO_3_^–^ and NO_2_^–^) in water using plasma technology, also known as plasma-activated water or plasma-treated water, represents a promising route for sustainable fertilizer production. In addition, plasma-activated water, being mildly acidic, can convert volatile NH_3_ into nonvolatile NH_4_^+^, and thereby reducing ammonia loss and atmospheric ammonia pollution.

A techno-economic analysis (TEA) by Rouwenhorst et al.^9^ suggests that if the EC for plasma-based NO_*x*_^–^ production could be reduced to 1.0–1.5 MJ mol^–1^, it would become competitive with the tandem e H–B and Ostwald process. Various plasma types, including DBD, GA, plasma jet, and spark discharge, have been adapted for NO_*x*_^–^ synthesis in liquids. Although DBD systems are generally inefficient in gas-phase NO_*x*_ formation, significantly lower ECs have been achieved in DBD-based liquid-phase systems, primarily due to enhanced plasma–liquid interactions.^115^

Beyond plasma type, the experimental configuration strongly affects the performance of plasma NO_*x*_^–^ production in liquid. Different experimental configurations have been explored to optimize NO_*x*_^–^ production, which can be broadly categorized into four configurations, as shown in Figure 11:^1^ water in plasma,^2^ plasma above water,^3^ plasma in water, and^4^ water trap after plasma. Table 1 summarizes the performance metrics and EC values of plasma-based NO_*x*_^–^ production using various plasma types and reactor configurations at atmospheric pressure.

**Figure 11:**
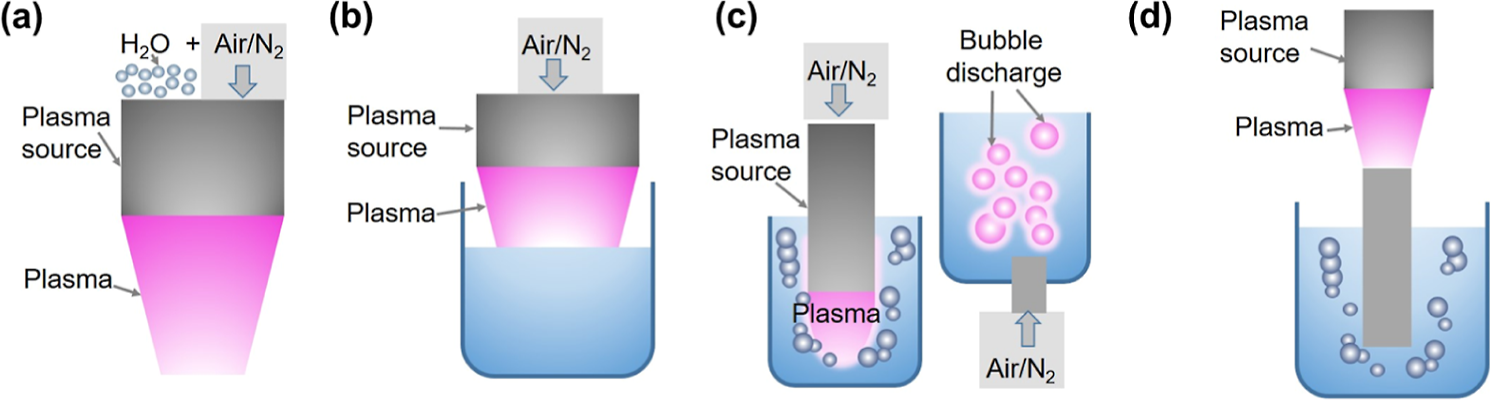
Experimental configurations of plasma-based NO_*x*_^–^ production in liquids:^1^ water in plasma, (b) plasma above water, (c) plasma in water, and (d) water trap after plasma.

**Table 1:** A Comparison of the Performance and EC of Plasma-Based NO_*x*_^–^ Production Using Various Plasma Types and Reactor configurations

plasma type	configuration	discharge power (W)	production rate (μmol h^–1^)	reactants in plasma	main products	energy consumption (MJ mol^–1^)	ref
spark-type plasma jet	air plasma with H_2_O vapor above water	0.1	14.25	air/water	NH_4_^+^, NO_3_^–^, NO_2_^–^, and H_2_O_2_	25.3	^116^
RGA-plasma jet	plasma jet above liquid	300	∼824.4	N_2_	NH_4_^+^, NO_3_^–^, and NO_2_^–^	∼1310	^117^
GA-type plasma jet	plasma jet above liquid with UV radiation		3324	N_2_	NH_4_^+^, NO_3_^–^, and NO_2_^–^		^118^
in liquid plasma	in liquid bubble discharge	130	672	N_2_ in liquid water	NH_4_^+^, NO_3_^–^, and NO_2_^–^	696.4	^119^
DBD plasma	water-falling film DBD reactor	130	11,898	air/water	NO_3_^–^	39.6	^115^
pulsed discharge	in liquid bubble discharge	52.3	468	N_2_ in liquid water	NO_3_^–^, and NO_2_^–^	403.2	^120^
DBD plasma	plasma-water droplet reactor	24	∼44	air/water	NH_4_^+^, NO_3_^–^, and NO_2_^–^	1963.6	^121^
arc and DBD	air plasma and O_2_ plasma with water trapping	260	122,000	air/O_2_	NO_3_^–^, NO_2_^–^, and H_2_O_2_	∼8	^122^
GA	air plasma with water trapping	58.6	89.3	air	NO_3_^–^, and NO_2_^–^	2362	^123^
DBD	air plasma above water	1000	72,580	air	NO_3_^–^	49.6	^124^
pulsed discharge	in liquid bubble discharge	52.5	∼1000	N_2_/O_2_/water	NO_3_^–^	186	^120^
glow and spark discharge	double reactor glow and spark discharge with raschig rings in water	11.27	2970	air	NO_3_^–^, and NO_2_^–^	13.7	^125^
glow discharge	glow discharge in water	7.38	445	air		59.7	
spark discharge	spark discharge in water	9.22	800	air		41.5	
glow and spark discharge	glow and spark discharge in a single reactor immersed in water	10.67	1871	air		20.5	
GA	GA above liquid	210	9600	air	NO_3_^–^, and NO_2_^–^	84.3	^126^
hot arc	hot arc above water	150	16,718	air	NO_3_^–^, NO_2_^–^, and H_2_O_2_	32.3	^127^
GA	GA with water spray AC	120	1521	air		286.3	^128^
	GA with water spray pulsed	0.25	217	air		4.14	
GA	two electrodes above water	250	30,967	air	NO_3_^–^	29	^129^
	two electrodes above water	250	25,806	N_2_	NO_3_^–^	34.9	
	three electrodes above water	500	51,613	air	NO_3_^–^	34.9	
	three electrodes with water spray	500	13,197	air	NO_3_^–^, and NO_2_^–^	136.4	

#### 3.1.1 Water in Plasma

Water is introduced directly into the plasma zone as droplets or vapor (Figure 11a).^115,116,121^ The plasma-generated NO and NO_2_ dissolve in water, where they form NO_3_^–^ and NO_2_^–^ via subsequent reactions with hydroxyl radicals (OH) and hydrogen peroxide (H_2_O_2_). Water therefore plays a dual role—serving both as a medium for capturing in situ generated NO_*x*_ species ([Disp-formula eqR13] and [Disp-formula eqR14]) and as a source of reactive intermediates. Water vapor undergoes dissociation under plasma conditions to yield H and OH radicals ([Disp-formula eqR15]), which further react with NO and NO_2_ to produce HNO_2_ and HNO_3_ ([Disp-formula eqR16] and [Disp-formula eqR17]), thereby promoting nitrogen fixation into the aqueous phase. Among DBD plasma systems, a water-falling film reactor achieved the lowest reported EC of 39.6 MJ mol^–1^, outperforming several DBD-based setups for gas-phase NO_*x*_ generation. However, it remains unclear whether introducing water significantly enhances N_2_ conversion. Additionally, although GA is considered energy-efficient for gaseous NO_*x*_ production, its aqueous NO_*x*_^–^ synthesis requires ECs exceeding those of gas-phase systems by more than one order of magnitude.^128,129^ Interestingly, substituting air with N_2_ as the carrier gas resulted in only a minor decrease in aqueous NO_*x*_^–^ yield, suggesting limited gas–liquid interactions. In contrast, DBD plasmas can generate ozone, which extends nitrogen oxidation process by converting NO into NO_2_, NO_3_, and N_2_O_5_ ([Disp-formula eqR18]–[Disp-formula eqR20]). The latter two species, with higher Henry’s law constants, are more soluble and thus enhance NO_*x*_^–^ accumulation in water via reactions [Disp-formula eqR21] and [Disp-formula eqR22].

R13 $$NO\left(g\right)+NO_{2}\left(g\right)+H_{2}O\left(l\right)\to 2{NO_{2}}^{-}\left(aq\right)+2H^{+}\left(aq\right)$$

R14 $$2NO_{2}\left(g\right)+H_{2}O\left(l\right)\to {NO_{3}}^{-}\left(aq\right)+{NO_{2}}^{-}\left(aq\right)+2H^{+}\left(aq\right)$$

R15 $$H_{2}O\left(g\right)+e^{-}\to H+OH+e^{-}$$

R16 $$NO\left(g\right)+OH\left(g\right)\to HNO_{2}\left(g\right)$$

R17 $$NO_{2}\left(g\right)+OH\left(g\right)\to HNO_{3}\left(g\right)$$

R18 $$NO\left(g\right)+O_{3}\left(g\right)\to NO_{2}\left(g\right)+O_{2}\left(g\right)$$

R19 $$NO_{2}\left(g\right)+O_{3}\left(g\right)\to NO_{3}\left(g\right)+O_{2}\left(g\right)$$

R20 $$NO_{2}\left(g\right)+{NO}_{3}\left(g\right)+M\to N_{2}O_{5}\left(g\right)+M$$

R21 $$NO_{3}\left(aq\right)+H_{2}O\left(l\right)\to 2H^{+}\left(aq\right)+{NO_{3}}^{-}\left(aq\right)+{OH}^{-}\left(aq\right)$$

R22 $$N_{2}O_{5}\left(aq\right)+H_{2}O\left(l\right)\to 2H^{+}\left(aq\right)+2N{O_{3}}^{-}\left(aq\right)$$

#### 3.1.2 Plasma Above Water

In the plasma-above-water configuration (Figure 11b), plasma is generated directly above the water surface, enabling vapor-phase H_2_O to enter the plasma and react with NO_*x*_ to form aqueous NO_*x*_^–^. Various plasma sources have been investigated, including DBD,^124,130,131^ plasma jets,^117,118^ glow^132^ and spark discharges,^133,134^ corona,^135^ hot arc,^127^ and GA.^126,128,129^ Jin et al.^124^ demonstrated enhanced NO_*x*_^–^ production using a cylindrical DBD setup with a water electrode, while Hoeben et al.^127^ achieved a high yield of 16,718 μmol h^–1^ using hot arc plasma. GA placed above the water surface reduced EC but led to NO_*x*_ loss, with NO_3_^–^ as the dominant product.^129^ In contrast, when N_2_ was used as the feed gas under UV irradiation, NO_2_^–^ became the major product.^118^ UV light promoted the formation of OH and H radicals from H_2_O, enhancing NO_*x*_^–^ production via secondary reactions (Figure 12). Interestingly, UV irradiation selectively promoted NO_2_^–^ formation, tripling its synthesis rate while increasing NO_3_^–^ formation by only 1.3 times. Although N_2_ was used as the feed gas, a noticeable amount of gaseous NO_*x*_ emission was still detected.

R23 $$H_{2}O\left(l\right)\overset{UV}{\to }H\left(aq\right)+OH\left(aq\right)$$

**Figure 12:**
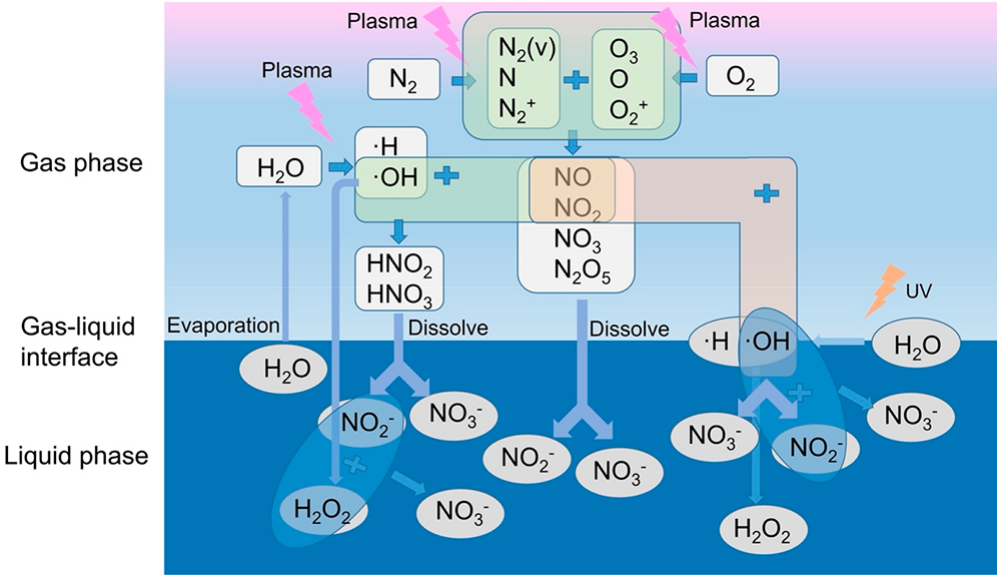
Schematic diagram of mechanisms of plasma-based NO_*x*_^–^ production in plasma above liquid configuration.

#### 3.1.3 Plasma in Water

To improve NO_*x*_ capture, some studies ignited plasma beneath the water surface (“plasma in water” configuration), including discharge-in-bubbles and direct plasma immersion systems. The bubble discharge offers a large interfacial area, enhancing gas–liquid transfer. Bian et al.^120^ employed pulsed discharges in bubbles to fix nitrogen, producing NO_3_^–^ and NO_2_^–^ with an EC of 186 MJ mol^–1^. NO_2_^–^ was subsequently converted to NO_3_^–^ by reactions with OH and H_2_O_2_ (R24 and R25), forming HNO_3_.

R24 $$N{O_{2}}^{-}\left(aq\right)+2OH\left(aq\right)\to N{O_{3}}^{-}\left(aq\right)+H_{2}O\left(l\right)$$

R25 $${{NO}_{2}}^{-}\left(aq\right)+H_{2}O_{2}\left(aq\right)\to N{O_{3}}^{-}\left(aq\right)+H_{2}O\left(l\right)$$

A recent study^125^ demonstrated that spark discharges in bubbles lowered breakdown voltage and produced reactive species more efficiently than glow discharges. Spark discharges in those bubbles provided a highly reactive environment for NO_*x*_ formation, whereas glow discharges in the tubular reactor region primarily activated N_2_ and O_2_ to generate excited N, N_2_ (vibrational states), O, O_2_, and O_3_ species along with minor NO_*x*_. As a result, NO_3_^–^ dominated in glow discharges with a selectivity of >90%, while NO_2_^–^ was more favored in spark discharges. Combining both discharge modes in a single configuration minimized energy losses thus reducing EC. A further reduction in EC to 13.7 MJ mol^–1^ was as achieved by adding Raschig rings into the water, which increased the gas–liquid interfacial area, enhanced mass transfer, and promoted the dissolution of gaseous NO_*x*_ species into the solution.

#### 3.1.4 Post-plasma Water Trapping

Besides generating plasma directly in water, a more practical approach to enhance gas–liquid interaction involves bubbling plasma-generated gases through water. Yang et al.^123^ employed a GA reactor coupled with a water-filled gas absorber for NO_*x*_^–^ production. They found that low-frequency discharges (50 Hz) required three times more energy than high-frequency ones (5–80 kHz), likely because higher frequencies produced a larger plasma volume. However, even under optimal conditions, the EC (2847 MJ mol^–1^) remained extremely high, largely due to the low solubility of NO (Henry’s law constant: 1.9 × 10^–5^ mol m^–3^ Pa^–1^) and NO_2_ (1.2 × 10^–4^ mol m^–3^ Pa^–1^) in water. One solution to address this limitation is to use more reactive liquids to trap NO_*x*_. Birkeland et al. demonstrated that alkali and alkaline earth hydroxide solutions can effectively trap NO_*x*_ and convert it to NO_3_^–^ with lower EC (3.4–4.1 MJ mol^–1^), though the multistage process is operationally complex.^24^ A simpler and highly effective hybrid approach was developed by Dinh,^122^ combining a GA reactor with a DBD-based ozone source. This hybrid plasma system converted gaseous NO_*x*_ and O_3_ into highly soluble NO_3_^–^ and N_2_O_5_ (Henry’s constants: 3.8 × 10^–4^ and 2.1 × 10^–2^ mol m^–3^ Pa^–1^), achieving nearly zero gas emissions and lowering the EC to 8 MJ mol^–1^. This design offers a promising route for direct NO_*x*_^–^ synthesis in water toward decentralized fertilizer production.

### 3.2 Plasma-Based Ammonia Production from Aqueous NO_*x*_^–^

Currently, most plasma-based ammonia production studies have focused on direct synthesis from N_2_ and H_2_.^136–145^ Considerable progress has been made using different plasma reactor configurations and catalysts to reduce the EC of NH_3_ production. However, recent studies indicate that this synthesis route suffers from a trade-off between low EC and high ammonia concentration.

Several groups^116,118,146–149^ have reported plasma-based ammonia synthesis from N_2_ and water. This route is particularly attractive as it eliminates the dependence on H_2_. So far, high ammonia production rates up to 2.55 mg h^–1^ ^118^ and ammonia selectivity approaching 100%^146^ have been reported using UV irradiation-enhanced plasma and plasma-assisted electrolytic system, respectively. However, the EC of ammonia production using these processes remains on the order of 100 MJ mol^–1^,^116,146,147^ far above that of the H–B process (0.48 MJ mol^–1^). This high EC could be due to the limited reaction at the plasma–liquid interface. In addition, reactive species such as atomic N and vibrationally excited N_2_ have extremely short lifetimes (∼10 ms),^150–152^ leading to significant energy loss if they fail to reach the interface before deactivation.

**Figure 13:**
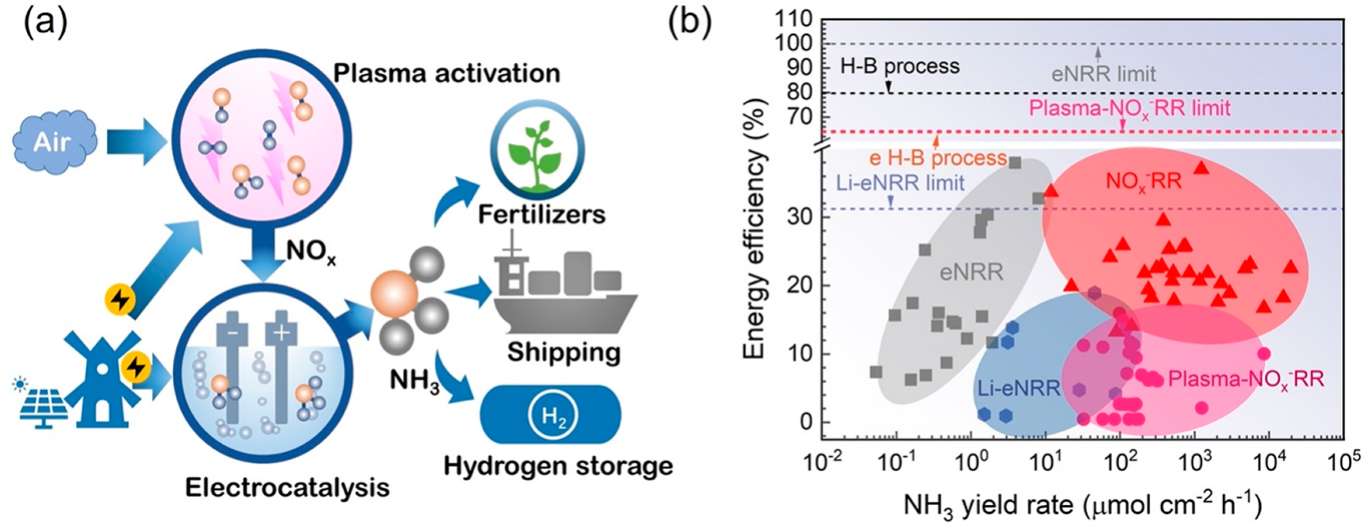
(a) Schematic diagram of plasma-assisted NO_*x*_^–^ reduction reaction for ammonia production and (b) ammonia equivalent production rate and energy efficiency for NO_*x*_^–^RR, plasma-NO_*x*_^–^RR, Li-mediated nitrogen reduction reaction (Li-eNRR), and electrocatalytic nitrogen reduction reaction (eNRR).

Recently, several studies have proposed using aqueous NO_*x*_^–^ as an intermedium for subsequent reduction to NH_3_, either catalytically^153,154^ or electrocatalytically.^125,126^ The combination of the NO_*x*_^–^ production process with electrolysis is particularly attractive as both processes operate at low temperatures and ambient pressure (Figure 13a).^155^ Compared with the inertness and low solubility of N_2_ in conventional electrolysis, NO_*x*_ species offer distinct advantages, including high solubility in water and low dissociation energy of the N=O bond. Moreover, NO_*x*_ is relatively stable compared to plasma-generated excited species, ensuring that the energy used for its production can be effectively utilized, thereby offering a potentially energy-efficient pathway for green ammonia synthesis.

Sun et al.,^125^ demonstrated plasma-assisted NO_*x*_^–^ reduction reaction (Plasma-NO_*x*_^–^RR) for ammonia production using a scalable electrolyzer. A plasma bubble reactor (“plasma-in-water” configuration) was used to produce NO_*x*_^–^, generating 0.4 mmol NO_*x*_^–^ in 100 mL water after 0.5 h of plasma activation. The selectivity toward NO_3_^–^ in NO_*x*_^–^ was ∼75%, so the main electrocatalytic reduction proceeded via R26, with the overall reaction described by R27. As eight electrons are required to reduce NO_3_^–^ to NH_4_^+^, but only six for NO_2_^–^ (R28), using NO_2_^–^ as the nitrogen source can improve energy efficiency. In subsequent electrocatalytic NO_*x*_^–^ reduction reaction (NO_*x*_^–^RR), Cu nanowires were used as electrocatalysts. Acidic media with an H_2_SO_4_ concentration of 10 mM yielded the highest ammonia production, while NO_2_^–^ feed generally resulted in higher Faradaic efficiency (FE) than NO_3_^–^, likely because NO_3_^–^ reduction to NO_2_^–^ (R29) competes with the desired NO_*x*_^–^RR and the hydrogen evolution reaction (HER, R30). Under optimal conditions, the EC for ammonia production (∼15.5 MJ mol^–1^) was mainly attributed to NO_*x*_^–^ formation (13.7 MJ mol^–1^).

R26 $${{NO}_{3}}^{-}\left(aq\right)+10H^{+}+8e^{-}\to {{NH}_{4}}^{+}\left(aq\right)+3H_{2}O\left(l\right)$$

R27 $$N{O_{3}}^{-}\left(aq\right)+H_{2}O\left(l\right)+2H^{+}\left(aq\right)\to N{H_{4}}^{+}\left(aq\right)+2O_{2}\left(g\right)$$

R28 $${{NO}_{2}}^{-}\left(aq\right)+8H^{+}\left(aq\right)+6e^{-}\to {{NH}_{4}}^{+}\left(aq\right)+2H_{2}O\left(l\right)$$

R29 $$N{O_{3}}^{-}\left(aq\right)+2H^{+}\left(aq\right)+2e^{-}\to {{NO}_{2}}^{-}\left(aq\right)+H_{2}O\left(l\right)$$

R30 $$2H^{+}+2e^{-}\to H_{2}$$

R31 $${{NO}_{2}}^{-}\left(aq\right)+6H_{2}O\left(l\right)+6e^{-}\to {{NH}_{4}}^{+}\left(aq\right)+8{OH}^{-}\left(aq\right)$$

R32 $${{NO}_{3}}^{-}\left(aq\right)+7H_{2}O\left(l\right)+8e^{-}\to {{NH}_{4}}^{+}\left(aq\right)+10{OH}^{-}\left(aq\right)$$

Other plasma configurations, such as “plasma above water” and “post-plasma water trapping”, have been explored to enhance NO_*x*_^–^ production rate and lower EC. Wu et al.^126^ placed a RGA above the liquid surface to produce NO_*x*_^–^; however, only a limited of the NO_*x*_ was converted to aqueous NO_*x*_^–^. The predominant aqueous products were NO_2_^–^ with a selectivity of >99% due to the use of an alkaline trapping solution. Therefore, ammonia was mainly produced via NO_2_^–^ reduction (R31) rather than NO_3_^–^ reduction (R32). The EC for ammonia production under optimum conditions was 85 MJ mol^–1^, which could be reduced to ∼3.2 MJ mol^–1^ if all gaseous NO_*x*_ were effectively captured in solution. Other studies^156,157^ using “post-plasma water trapping” configurations also aimed to trap gaseous NO_*x*_, but their spark and DBD systems likely exhibited much higher ECs for NO_*x*_ production (8.7 MJ mol^–1^ for spark discharge and 15.9 MJ mol^–1^ for DBD), as shown in Figure 4.

To enhance ammonia yield and FE, various electrocatalysts such as Co single atoms (SAs)/N–C, Ni_3_B@NiB_2.74_, and Cu nanoparticles have been investigated. Optimized operating conditions enabled FE exceeding 90%. For example, Li et al.^156^ reported nearly 100% FE using Ni_3_B@NiB_2.74_ as the electrocatalyst at an applied potential of 0.3 V vs RHE and NO_3_^–^ concentration of 100 mM. The NO_*x*_^–^ concentration also plays a crucial role—higher NO_*x*_^–^ levels generally favor higher NH_3_ production rates and FEs, regardless of the electrocatalyst type.

Figure 13b compares ammonia production rates and energy efficiencies across different ammonia synthesis technologies. The energy efficiency (η) is defined as the ratio between the energy content of the produced ammonia, *E*_out_, to the total energy input to the system for ammonia synthesis, *E*_in_

$$\eta =\frac{E_{out}}{E_{in}}=\frac{\Delta E_{out}}{\Delta E_{in}}=\frac{HHV}{\Delta E_{in}}$$

Manthiram et al.^158^ define Δ*E*_out_ as the free energy required to oxidize 1 mol of NH_3_ to H_2_O and N_2_ (Δ*H*_R_^0^ = 339 kJ mol^–1^). This academic approach differs from industrial practice, where either the higher heating value (HHV) or the lower heating value (LHV) of the fuel is typically used, depending on the specific application. For ammonia, the HHV is more appropriate owing to its high hydrogen content, which leads to substantial water formation during combustion in power plants and condensing boilers, enabling practical heat recovery from water vapor. The HHV of ammonia (382.8 kJ mol^–1^) represents the total heat released during complete combustion of ammonia to form nitrogen and liquid water under standard conditions.

As illustrated in Figure 13b, plasma-NO_*x*_^–^RR achieves a competitive ammonia production rate but currently exhibits lower energy efficiency than Li-mediated electrocatalytic nitrogen reduction (Li-eNRR) and electrocatalytic nitrogen reduction (eNRR). Since most of the energy input (>10 MJ mol^–1^) originates from NO_*x*_^–^ production rather than electrolysis (∼1.5 MJ mol^–1^), reducing the EC of NO_*x*_^–^ formation is critical to improving overall performance. Theoretical analysis suggests that the maximum energy efficiency of plasma-NO_*x*_^–^RR (64.6%) can exceed that of the e H–B process (63.8%) and Li-eNRR (31.2%).^159^

## 4 Outlook and Future Perspectives

Plasma-enabled NO_*x*_ synthesis has advanced significantly, evolving from exploratory investigations toward a more comprehensive understanding that integrates experiments and modeling to elucidate dominant reaction pathways under realistic operating conditions. These insights have driven significant progress in reactor engineering, plasma–catalyst integration, and plasma–liquid interface design. Despite these developments, the EC of plasma-based NO_*x*_ synthesis remains higher than that of the benchmark e H–B process combined with the Ostwald process. Therefore, further performance improvements are crucial, such as optimizing reactor design to enhance gas–plasma interactions, and ensuring that a larger fraction of gas passes through the discharge region. Energy efficiency can also be increased by suppressing backward reactions and reducing VV relaxation between N_2_ and O_2_. Fast quenching and in situ removal of NO (e.g., rapid oxidation of NO to NO_2_ by O_3_) help to reduce reverse reactions, while while preheating the feed gas can limit VV relaxation by facilitating thermal O_2_ dissociation and vibrational excitation of N_2_. Further research is needed to evaluate the practical implementation of these strategies. In addition, the scarcity of pilot-scale developments on plasma-based NO_*x*_ synthesis limits guidance for industrial translation. To advance commercial deployment, future efforts should prioritize scale-up demonstrations and comprehensive techno-economic assessments.

Coupling catalysts with plasma systems represents another promising direction for increasing NO_*x*_ yield and reducing EC. To date, most plasma-catalytic NO_*x*_ studies have been performed in DBD reactors, which are inherently limited by low energy efficiency and high EC. Recent studies suggest that combining GA and their variants with catalysts could substantially reduce EC. Future research should focus on the rational design of high-performance plasma-catalytic systems and the development of cost-effective catalysts that remain active under nonequilibrium plasma conditions. The underlying mechanisms governing plasma-catalyst interactions in NO_*x*_ synthesis remain poorly understood, particularly which plasma-activated N_2_ species dominate NO_*x*_ formation and which catalytic sites contribute to plasma-enhanced reactions. Addressing these knowledge gaps will require advanced in situ and operando diagnostics, including in situ plasma-coupled FTIR, in situ Raman, laser-based spectroscopic techniques, coupled with complementary modeling approaches. Together, these tools can provide molecular-level insights into plasma-catalyst interactions beyond what can be inferred from macroscopic observations, informing system-level design for successful process scale-up.

In addition to gas-phase NO_*x*_, nitrogen fixation via direct and indirect aqueous NO_*x*_^–^intermediates represents a complementary pathway, forming a tandem plasma-electrocatalytic process. Direct routes involve plasma generation in water, while indirect routes rely on gas-phase NO_*x*_ dissolving into water. These aqueous intermediates offer higher solubility, lower dissociation energy of the N=O bond, and greater stability than excited N and N_2_ species, enabling more robust conversion to ammonia compared with conventional eNRR under ambient conditions. Future work should aim to improve the energy efficiency of aqueous NO_*x*_^–^generation, optimize reactor configurations to enhance gas–liquid mass transfer, and develop robust, cost-effective and high-activity electrocatalysts for NO_*x*_^–^ reduction. Systematic techno-economic and life-cycle assessments, as well as pilot-scale demonstrations, are essential to evaluate practical viability.

While plasma-assisted nitrogen fixation is generally regarded as an environmentally benign route, several challenges remain, particularly the formation and management of undesired NO_*x*_ effluents and byproducts such as O_3_ and N_2_O. These issues can be effectively mitigated through multiple strategies. NTP systems, including glow discharge, GA, and MW plasmas, typically operate at elevated gas temperatures. This intrinsic thermal condition promotes the decomposition of O_3_, thereby limiting its accumulation during plasma-based NO_*x*_ production. In addition, optimizing discharge parameters, tailoring the N_2_/O_2_ ratio, and introducing suitable catalysts downstream can promote the selective formation of NO_*x*_ (NO and NO_2_) while minimizing side reactions, such as N_2_O formation. Efficient gas–liquid contact and repeated absorption of NO_*x*_ into water to produce nitric or nitrous acids effectively prevent atmospheric emissions, enabling near-complete nitrogen utilization in a closed-loop system. In this process, controlling the NO_2_/NO ratio is particularly important, as NO_2_ is more readily absorbed into water, facilitating efficient nitric acid formation, and minimizing gaseous NO_*x*_ release.

Moving forward, plasma-based NO_*x*_ synthesis offers unique advantages that align closely with the transition to a net-zero economy. Its ability to start and stop instantly, flexibility with intermittent renewable power, modular design, and use of abundant, low-cost feedstock such as air make it ideally suited for decentralized, small-to medium-scale fertilizer production. By enabling production near farms, these systems can reduce CO_2_ emissions associated with transporting fertilizers from centralized H−B plants. Such decentralized systems could complement, rather than replace, centralized H−B plants, creating a hybrid nitrogen economy that couples large-scale efficiency with distributed resilience. Realizing this vision will require more than scientific breakthroughs: techno-economic and life-cycle assessments, integration with renewable energy grids, and supportive policy frameworks will be essential for practical, sustainable deployment.

In summary, while plasma-based NO_*x*_ synthesis still faces significant efficiency and scale-up challenges, it represents a genuinely transformative pathway toward sustainable nitrogen fixation. By advancing reactor-catalyst integration, deepening mechanistic understanding, demonstrating pilot-scale feasibility, and embedding techno-economic and policy considerations, plasma-driven nitrogen fixation could evolve from laboratory curiosity to an industrially relevant process. With continued interdisciplinary innovation, it has the potential to redefine nitrogen chemistry and contribute significantly to sustainable agriculture and global decarbonization. Plasma technology could ultimately electrify nitrogen fixation for the 21st century, enabling fertilizers to be produced directly from air, water, and renewable energy sources.
